# EZH2-induced lysine K362 methylation enhances TMPRSS2-ERG oncogenic activity in prostate cancer

**DOI:** 10.1038/s41467-021-24380-6

**Published:** 2021-07-06

**Authors:** Marita Zoma, Laura Curti, Dheeraj Shinde, Domenico Albino, Abhishek Mitra, Jacopo Sgrignani, Sarah N. Mapelli, Giada Sandrini, Gianluca Civenni, Jessica Merulla, Giovanna Chiorino, Paolo Kunderfranco, Alessia Cacciatore, Aleksandra Kokanovic, Andrea Rinaldi, Andrea Cavalli, Carlo V. Catapano, Giuseppina M. Carbone

**Affiliations:** 1 Università della Svizzera italiana (USI), Institute of Oncology Research (IOR), Bellinzona, Switzerland; 2 Università della Svizzera Italiana (USI), Institute for Research in Biomedicine (IRB), Bellinzona, Switzerland; 3 Swiss Institute of Bioinformatics (SIB), Lausanne, Switzerland; 4 Laboratory of Cancer Genomics, Fondazione Edo ed Elvo Tempia Valenta, Biella, Italy; 5 Present Address: Center for Genomic Science of IIT@SEMM, Fondazione Istituto Italiano di Tecnologia (IIT), Milan, Italy; 6 Present Address: Humanitas Research Center, Milan, Italy

**Keywords:** Prostate cancer, Epigenetics, Oncology

## Abstract

The TMPRSS2-ERG gene fusion is the most frequent alteration observed in human prostate cancer. However, its role in disease progression is still unclear. In this study, we uncover an important mechanism promoting ERG oncogenic activity. We show that ERG is methylated by Enhancer of zest homolog 2 (EZH2) at a specific lysine residue (K362) located within the internal auto-inhibitory domain. Mechanistically, K362 methylation modifies intra-domain interactions, favors DNA binding and enhances ERG transcriptional activity. In a genetically engineered mouse model of ERG fusion-positive prostate cancer (*Pb-Cre4 Pten* ^*flox/flox*^ *Rosa26-ERG, ERG/PTEN*), ERG K362 methylation is associated with PTEN loss and progression to invasive adenocarcinomas. In both ERG positive VCaP cells and ERG/PTEN mice, PTEN loss results in AKT activation and EZH2 phosphorylation at serine 21 that favors ERG methylation. We find that ERG and EZH2 interact and co-occupy several sites in the genome forming trans-activating complexes. Consistently, ERG/EZH2 co-regulated target genes are deregulated preferentially in tumors with concomitant ERG gain and PTEN loss and in castration-resistant prostate cancers. Collectively, these findings identify ERG methylation as a post-translational modification sustaining disease progression in ERG-positive prostate cancers.

## Abstract (web summary)

Although the TMPRSS2-ERG gene fusion is the most common alteration in human prostate cancer, its involvement in disease progression remains unclear. Here, the authors demonstrate that ERG is methylated by Enhancer of zest homolog 2 leading to enhanced transcriptional and oncogenic activity.

## Introduction

ETS transcription factors constitute a family of signal-dependent transcriptional regulators controlling cell proliferation, differentiation, and carcinogenesis^1^. Gene fusions involving the ETS factor ERG and the androgen-regulated serine protease TMPRSS2 are found in about half of prostate tumors and represent one of the most frequent genetic rearrangements in human cancers^2,3^. The TMPRSS2–ERG fusion provides a mechanism for androgen-induced ERG overexpression and reprogramming of the transcriptome of prostate epithelial cells^4–6^. However, the mechanisms of progression in tumors with aberrant expression of ERG is still unclear^7^. ERG requires additional cooperating factors to exert its oncogenic effects. In prostate cancer patients, ERG gene fusion is frequently concomitant with PTEN loss and both events are associated with more aggressive disease^8^. Consistently, in genetically engineered mouse models (GEMMs), ERG transgenic mice develop pre-neoplastic lesions and invasive lesions only when they are crossed with PTEN-deficient mice^8–10^. Thus, crosstalk with multiple signaling pathways are apparently required for progression of ERG-fusion positive tumors. Understanding the underlying mechanisms may reveal new prognostic and therapeutic tools that may have a relevant impact for the management of prostate cancer patients.

In this study, we provide a new paradigm for ERG oncogenic activation in the context of ERG fusion positive tumors. We find that Enhancer of zest homolog 2 (EZH2), the histone H3K27 methyltransferase (MT) within the Polycomb repressive complex 2 (PRC2)^11^, interacts with ERG and catalyzes methylation of ERG at lysine 362 (K362) which enhances its transcriptional and oncogenic activity. Mechanistically, K362 methylation results in a conformational switch disrupting the internal ERG auto-inhibitory domain, promoting DNA binding and enhancing transcription of ERG target genes. ERG K362 methylation is associated with stem-like, tumorigenic, and metastatic properties in cell lines and with progression from non-invasive lesions to invasive adenocarcinomas in ERG/PTEN mice. Both in cell lines and mouse models, PTEN loss leads to enhanced EZH2-mediated ERG methylation throughout AKT activation and EZH2 phosphorylation, providing a mechanistic explanation for the cooperation between ERG gain and PTEN loss. Consistently, we find that ERG/EZH2 co-regulated genes are transcriptionally active preferentially in ERG-positive primary tumors with concomitant PTEN deficiency and in castration-resistant prostate cancer (CRPC) concomitantly overexpressing ERG and EZH2, underlying the clinical relevance of these findings. Thus, K362 lysine methylation identified in this study is a relevant post-translational modification of ERG that sustains disease progression in ERG-positive tumors and provides rationale for developing alternative therapeutic strategies to antagonize ERG oncogenic activity.

## Results

### ERG is methylated in ERG fusion positive prostate cancer cells

**Fig. 1:**
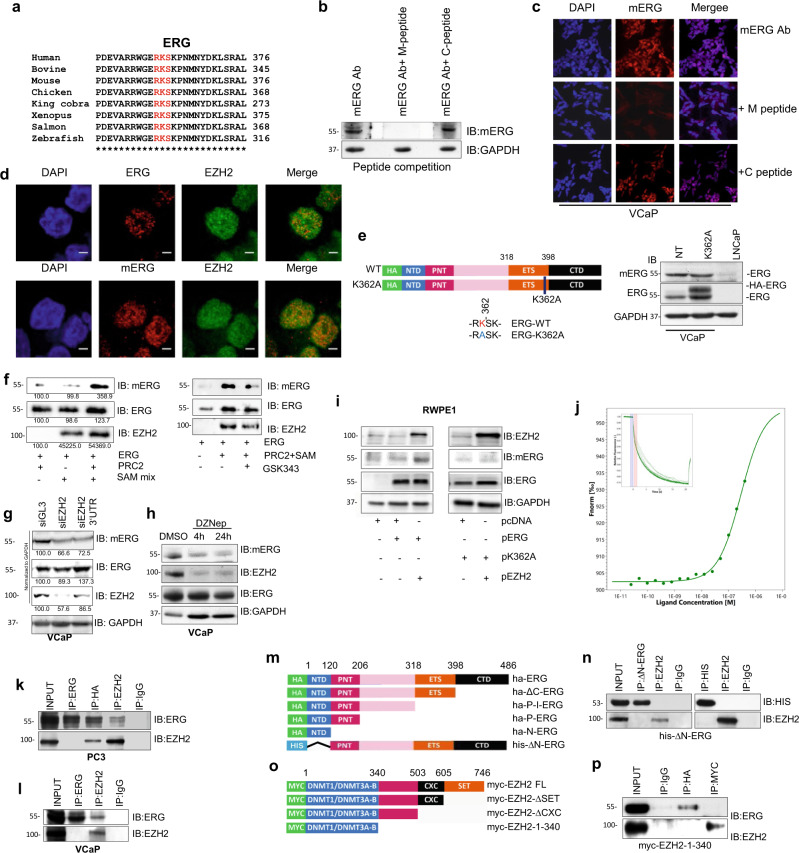
EZH2 methylates ERG at lysine K362. **a** Sequence alignment of ERG domain containing the EZH2 recognized R-K-S motif from diverse species. **b** Detection of methylated ERG in VCaP cells by immunoblotting with anti-mERG antibody and competition with methylated (M) and non-methylated (C) peptides (*n* = 2). **c** Detection of mERG in VCaP cells by immunofluorescence microscopy with anti-mERG antibody pre-incubated with the specific competitor and control peptides (*n* = 2). Scale bar = 20 µm. **d** Detection of ERG, EZH2, and mERG in VCaP cells by immunofluorescence microscopy (*n* = 2). Scale bar = 20 µm. **e** Detection of ERG and mERG by IB in control and HA-tagged K362A ERG transfected VCaP cells (*n* = 2). **f** In vitro methylation assay with recombinant ERG and EZH2 followed by immunoblots with indicated antibodies (left) and in the presence of the EZH2 inhibitor GSK343 (right) (*n* = 2). **g** Detection of mERG, ERG, and EZH2 by IB in VCaP cells upon EZH2 knockdown by two siRNA (siEZH2 and siEZH2 3′UTR) (*n* = 2). **h** Detection of mERG, ERG, and EZH2 by IB in VCaP cells upon treatment with 10 µM DZNep (H) at indicated time points (*n* = 2). **i** Immunoblots of mERG, ERG, and EZH2 in RWPE1 cells transiently transfected with the indicated ERG and EZH2 expression vectors (*n* = 2). **j** Binding of recombinant ERG and EZH2 determined by microscale thermophoresis (MST). Insert, MST tracing. **k** Co-IP of ERG and EZH2 in PC3 cells transiently transfected with Ha-tagged ERG expression vector (*n* = 2). **l** Co-IP of ERG and EZH2 in VCaP cells with ERG and EZH2 specific antibodies and control IgG (*n* = 2). **m** Diagram of truncated ERG constructs. **n** Co-IP and His-pulldown in PC3 cells transiently transfected with the His-ΔN-ERG constructs and immunoblotting with anti-His and anti-EZH2 antibodies (*n* = 2). **o** Diagram of truncated EZH2 constructs. **p** Binding of Myc-EZH2-∆SET to Ha-ERG assessed by co-immunoprecipitation in PC3 cells transiently transfected with the truncated EZH2 constructs along with ERG plasmid (*n* = 2). Molecular weights are indicated in kilodaltons (kDa). Source data are provided as a Source Data File.

In addition to canonical PRC2-dependent activity, EZH2 exerts non-PRC2 dependent functions through interaction and methylation of non-histone proteins^12–15^. We discovered that ERG contains an evolutionary conserved histone H3K27-like EZH2 recognition motif (R-K-S) centered at lysine 362 (K362) (Fig. 1a). To detect K362 methylated ERG (mERG) we generated a rabbit polyclonal antibody against a K362 mono-methylated ERG peptide. Using an ELISA assay with immobilized biotinylated ERG peptides, we demonstrated the high affinity of the newly generated antibody for mono-methylated peptide and very low affinity for non-methylated, and di- and tri-methylated peptides (Supplementary Fig. 1a). We tested the antibody activity and specificity also by immunoblotting. Consistently, the anti-mERG antibody detected only the mono-methylated form of the ERG peptide (Supplementary Fig. 1b); neither the non-methylated nor di- and tri-methylated peptides were detected, confirming the ability of the antibody to specifically detect the mono-methylated form of ERG. Immunoblotting (Fig. 1b) and immunofluorescence microscopy (Fig. 1c) in the presence of methylated (M-peptide) and non-methylated (C-peptide) competitor peptides confirmed the specifity of the signal detected with the anti-mERG antibody in both cell lysates and intact ERG fusion positive VCaP cells. Furthermore, in VCaP cells mERG localized to the nucleus, similar to total ERG and EZH2 (Fig. 1d). We generated also an ERG construct with mutated K362 (HA-ERG-K362A) to prevent methylation (Fig. 1e). Notably, the anti-mERG antibody did not react with the ectopically expressed HA-tagged ERG-K362A mutant while clearly detected the endogenous mERG (Fig. 1e). Furthermore, no signal of mERG and ERG was detected in ERG-negative LNCaP cells. Immunoprecipitation with an anti-ERG antibody followed by immunoblotting with an anti-methyl lysine antibody further demonstrated that methylation of ERG occurred in VCaP cells (Supplementary Fig. 1c).

### EZH2 induces methylation of ERG at lysine K362

To determine whether EZH2 catalyzed ERG methylation, we performed in vitro methylation assays with recombinant ERG and purified PRC2 complex. Methylated ERG was detected by immunoblotting with the anti-mERG antibody in the complete reactions and was not seen in the absence of PRC2 and S-adenosylmethionine (SAM) or in the presence of GSK343, a catalytic inhibitor of EZH2 (Fig. 1f). Interestingly, recombinant EZH2 alone was able to catalyze methylation of purified ERG similarly to the full PRC2 complex (Supplementary Fig. 1d) in line with the notion that EZH2 is sufficient for mono-methylation of many non-histone proteins^13^.

In support of the EZH2 role in ERG methylation, knockdown of EZH2 by siRNAs reduced mERG in VCaP cells (Fig. 1g). Furthermore, the effect of EZH2 knockdown using a 3′UTR-targeting siRNA was rescued by ectopic expression of WT-EZH2 and not of the catalytic inactive EZH2-∆SET mutant (Supplementary Fig. 1e). DZNep, a known inhibitor of EZH2, reduced mERG along with total EZH2 similar to siRNA-mediated EZH2 knockdown (Fig. 1h). On the other hand, in immortalized prostate epithelial RWPE1 cells ectopically expressed WT-ERG was methylated only in the presence of EZH2 and the HA-ERG-K362A mutant was not methylated (Fig. 1i), in line with the requirement of both EZH2 activity and intact K362 for ERG methylation. Collectively, these results identified mono-methylation of K362 as a relevant post-translational modification of ERG induced by EZH2.

### ERG and EZH2 interact physically

Based on the evidence of their functional interaction, we examined whether ERG and EZH2 interacted physically. To test this, we performed microscale thermophoresis and detected binding of purified recombinant ERG and EZH2 proteins (*K*_*d*_ = ~290 nM) (Fig. 1j). We performed also immuno-precipitation in PC3 cells expressing endogenous EZH2 and ectopic HA-tagged ERG and using the anti-EZH2 and anti-HA antibodies we confirmed their interaction (Fig. 1k). IP with EZH2 antibody confirmed the binding to endogenous ERG in VCaP cells (Fig. 1l). However, using the anti-ERG antibody we did not detect co-immunoprecipitation of EZH2, likely due to the masking of the antibody epitope in the ERG N-terminal domain (NTD) by the ERG–EZH2 complex, although the anti-ERG antibody co-immunoprecipitated AR, a known ERG interacting protein^8^ (Supplementary Fig. 1f).

Next, to verify the region of ERG interacting with EZH2, we carried out immunoprecipitation with truncated HA-tagged ERG constructs (Fig. 1m and Supplementary Fig. 2a, b). All the ERG constructs retaining the NTD-bound EZH2 (Supplementary Fig. 2c–f). Conversely, the His-ΔN-ERG lacking the NTD failed to do so (Fig. 1n), indicating that the NTD was essential for ERG–EZH2 interaction. This finding is in line with recent data mapping the EZH2 interacting domain to the N-terminal region of the protein^16^. Interestingly, the TMPRSS2:ERG truncated variants (Δ32), expressed in VCaP cells, retains the ability to bind EZH2 and to be methylated (Fig. 1l and Supplementary Fig. 2g). This is also consistent with other studies^16,17^. To define the interacting domain in EZH2, we used truncated Myc-tagged EZH2 constructs (Fig. 1o and Supplementary Fig. 2h, i). Myc-EZH2-ΔSET and Myc-EZH2-ΔCXC interacted with ERG (Supplementary Fig. 2j, k), whereas Myc-EZH2-1-340 did not bind ERG (Fig. 1p). Thus, the region of EZH2 between residue 340 and 503 is required for interaction with ERG. Importantly, co-IP with the anti-EZH2 antibody and immunoblotting, showed that ERG and EZH2 interacted and was methylated in ERG fusion positive human primary prostate tumors (Supplementary Fig. 2l, m).

### K362 methylation modulates ERG protein conformation in the autoinhibitory domain

**Fig. 2:**
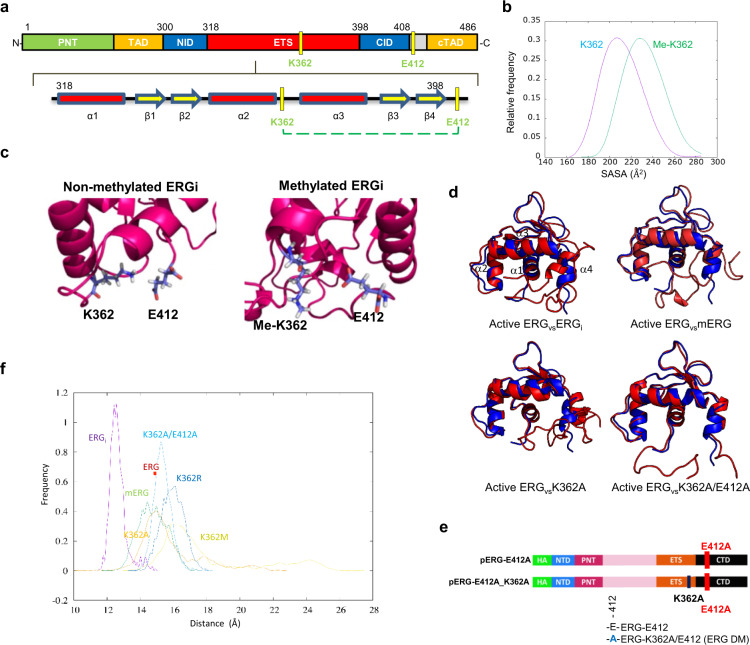
K362 methylation affects protein conformation. **a** ERG domain structure and organization of the ETS DNA binding domain and auto-inhibitory modules. **b** Solvent accessible surface area for non-methylated (K362) and methylated (Me-K362) form of the ERGi domain. **c** Representative structures from molecular dynamic simulation of non-methylated (left) and methylated (right) ERGi domain. **d** Overlay of ERG domain structure of active DNA-bound ERG derived from the X-ray model (blue ribbon) and structures of ERGi, methylated ERG, and indicated ERG mutants derived from molecular dynamic simulations. **e** Diagram of single (E412A) and double mutant (K362A/E412A) ERG constructs. **f** Inter-molecular distance in MD simulated ERG domain structures. The distance between residue Leu320 in α1 helix and Ala413 in α4 helix was calculated in all systems over the simulation time to estimate the degree of divergence relative to the active DNA-bound ERG (4iri) structure defined by X-ray crystallography. The same parameter measured in the X-ray structure identified by the pdb code 4iri is depicted as red square. ERGi (Violet), mERG (green), K362A/E412A (light blue) K362A (orange), K362M (yellow), and K362R (blue).

Trans-activation by ETS factors is regulated by internal auto-inhibitory domains^1^. ERG contains two auto-inhibitory modules, defined as N-terminal and C-terminal inhibitory domains (NID and CID), which flank the ETS domain and interfere with DNA binding through an allosteric mechanism^18^. K362 is located in the ETS domain within a short loop between the helices α2 and α3 (Fig. 2a). We hypothesized that methylation of K362 could affect the dynamic interactions of the ETS domain with the NID and CID auto-inhibitory modules. To test this hypothesis, we performed molecular dynamics simulation (MDS) using the available X-ray defined crystal structure of the ERG domain (ERGi) in the auto-inhibited conformation as starting point, to compare in silico the K362 methylated and non-methylated state^18^. MDS allows capturing dynamic changes in protein conformation that are difficult to interrogate with other experimental techniques. Interestingly, we found an increase of the solvent accessible surface area (SASA) for methylated K362 compared to the non-methylated K362 with an average area of 240 ± 18 Å^2^ and 218 ± 18 Å^2^, respectively (Fig. 2b). This increased accessibility was also evident by inspecting the MDS trajectories with methylated K362 residue pointing more towards the surface of the domain compared to the non-methylated K362 (Fig. 2c). Using MDS we examined also the dynamics of the interactions between K362 with other residues in the ETS domains, estimating the frequency of the specific contacts made by the methylated and non-methylated K362 during the time of simulation. Methylation of K362 affected multiple contacts with other amino acids (Supplementary Fig. 3a). Notably, we found that methylation disrupted the interaction of K362 with E412, a residue at the edge of the CID that is involved in the auto-inhibition of ERG (Fig. 2c).

Next, to gain more insights in the conformational changes induced by K362 methylation, we compared in silico the structures of active DNA-bound ERG^18^, autoinhibited ERGi, and mutated forms of ERG (Supplementary Fig. 3b). The auto-inhibited ERGi differed in many aspects from the active DNA-bound ERG, particularly in the position of the α4 helix that points towards the α1 helix limiting the access to DNA (Fig. 2d). Conversely, mERG overlaps closely the structure of the active DNA-bound ERG, with the α4 helix in a position more permissive for DNA binding. The K362A-ERG mutant differed from active ERG and mERG and assumed a conformation less favorable for DNA binding. Interestingly, the double mutant K362A/E412A-ERG (ERG DM), in which both K362 and E412 had been mutated in alanine eliminating any interaction between the two residues (Fig. 2e), reproduced closely the structure of the active DNA-bound ERG assuming a conformation favorable for DNA binding. The single E412A mutant recapitulated only partially the changes seen in the double mutant (Supplementary Fig. 3b). Thus, lysine methylation alters the relations of K362 with other amino acids in the ERG domain and induces secondary conformational changes that could positively influence ERG binding to DNA and its transcriptional activity. To estimate further the degree of structural divergence of the ERG variants from active DNA-bound ERG, we assessed the distance between two reference residues in the α1 (Leu320) and α4 (Ala413) helices (Fig. 2f). Using this approach, we found that ERGi was the most divergent from active ERG. In contrast, mERG was closer to WT ERG. Interestingly, the double mutant K362A/E412A was closer to active ERG and had a more stable structure than all the other variants, suggesting that it could mimic a constitutively uninhibited state of the protein. Among the K362 mutants, K362R and K362M diverged substantially from both active ERG and ERGi, in line with the broadly altered conformation of the two mutants (Supplementary Fig. 3b). Instead, the K362A mutant was closer to WT ERG and mERG indicating that the latter was a more conservative amino acid substitution to simulate the non-methylated state of ERG without major alterations of the overall structure. (Fig. 2f).

### Methylated K362 promotes ERG transcriptional and oncogenic activity

**Fig. 3:**
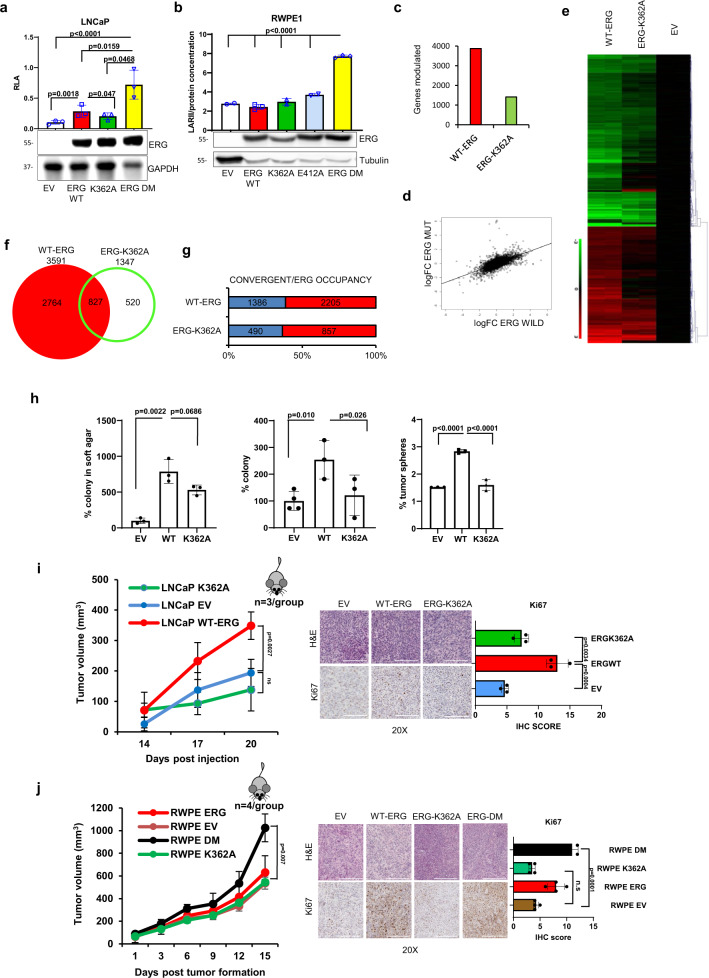
K362 methylation enhances ERG transcriptional and oncogenic activity. **a** Luciferase activity of the ETS responsive reporter in LNCaP cells expressing WT, K362A, K362A/E412A ERG, or empty vector (EV). Bottom, protein expression verified by immunoblotting. **b** Luciferase activity of the ETS responsive reporter in RWPE1 cells expressing WT, K362A, E412A, K362A/E412A ERG, or empty vector (EV). Bottom, protein expression verified by immunoblotting. **c** Total modulated genes in LNCaP cells expressing WT or K362A ERG. **d** Heat map showing the intensity of the transcripts significantly modulated in LNCaP cells expressing WT, K362A ERG, or empty vector (EV). Scale bar shows LogRatio range. Red, upregulation; green, downregulation (*n* = 2/group). **e** Scatter plot of the LogRatios of genes deregulated in LNCaP-ERG vs control (logFC ERG WILD) and LNCaP-ERG-K362A vs control (logFC ERG MUT). Regression line was calculated by means of the lm function (regression line coefficient = 0.44). **f** Venn diagram showing the convergence of genes modulated by WT-ERG and ERG-K362A. **g** Convergence between genes modulated by WT-ERG or ERG-K362A and ERG genomic occupancy in VCaP cells. **h** Soft agar, clonogenic, and sphere forming assays in LNCaP cells expressing WT, K362A ERG or an empty vector (EV). **i** Growth of xenografts of LNCaP cells expressing WT-ERG, ERG-K362A, or an empty vector (EV) injected in NSG mice (*n* = 3/group). Right, histological and Ki67 immunostaining scores in tumor xenografts. Scale bars represent 200 µm. **j** Growth of xenografts of RWPE1 cells expressing WT, ERG-K362A, K362A/E412A (DM) ERG, or an empty vector (EV) injected in NSG mice (*n* = 4, biological independent samples). Right, histological and Ki67 immunostaining scores in tumor xenografts. Scale bars represent 200 µm. All error bars, mean ± s.d. *P*-values were determined by one-way ANOVA test. Source data are provided as a Source Data File.

To evaluate the functional implication of our finding, we assessed the trans-activating ability of WT and ERG variants in luciferase reporter assays upon transfection in ERG-negative LNCaP and RWPE1 cells. When transfected in LNCaP cells, WT-ERG was methylated by EZH2, whereas the K362A-ERG mutant was not (Supplementary Fig. 4a). Consistently, WT-ERG and not the K362A-ERG mutant activated the ETS responsive reporter (Fig. 3a). Interestingly, the K362A/E412A double mutant (ERG DM) was considerably more active than both K362A and WT-ERG, in line with the hypothesis that the double mutant mimicked the uninhibited form of ERG. Notably, in RWPE1 cells WT-ERG, similar to the single mutants, was not active (Fig. 3b). Conversely, the ERG DM was able to induce the ETS responsive reporter in RWPE1 cells even in the absence of EZH2 co-expression (Fig. 3b), indicating that it acted as a uninhibited form of ERG and could overcome the need of EZH2-induced methylation. Consistently, the ERG DM was able to significantly induce the ETS promoter also in the presence of the EZH2 inhibitor GSK343 (Supplementary Fig. 4b). We verified also the ability of WT-ERG and mutant forms to bind to the promoter of known ERG target genes, IL-6 and PLAT1, in RWPE1 cells by chromatin immunoprecipitation (ChIP). The ERG DM mutant showed greater binding to the IL-6 and PLAT1 promoter compared to WT-ERG, whereas K362A-ERG showed reduced binding ability (Supplementary Fig. 4c). Thus, K362 methylation induced conformational changes that antagonized the auto-inhibitory modules and favored DNA binding and transcriptional activity. Next, to assess the impact of ERG methylation globally on the cell transcriptome, we compared the gene expression profiles of control (EV), WT-ERG and ERG-K362A expressing LNCaP cells. WT-ERG induce multiple changes involving both activated and repressed genes (Fig. 3c and Supplementary dataset 1). Expression of the methylation-defective ERG-K362A mutant resulted in a significantly reduced transcriptional response compared to WT-ERG, evident in terms of both number of genes (Fig. 3c) and fold change intensity (Fig. 3d–e), consistently with an ERG attenuated function. Nevertheless, genes modulated by ERG-K362A significantly overlapped with those affected by WT-ERG (Fig. 3f). Furthermore, genes modulated by both WT-ERG and ERG-K362A in LNCaP cells were mainly canonical ERG targets as shown by ChIP-sequencing in VCaP cells (Fig. 3g).

Preventing methylation by expressing K362A-mutated ERG resulted in attenuated ERG transcriptional activity. Notably, when stably expressed in LNCaP cells (Supplementary Fig. 4d), the ERG-K362A mutant exhibited also reduced ability compared to WT-ERG to promote oncogenic phenotypes, including anchorage-independent growth, colony formation, tumor-sphere formation in prostate-sphere assay, survival in anoikis, and cell migration (Fig. 3h and Supplementary Fig. 4e, f). The growth of tumor xenografts of LNCaP cells in mice was significantly enhanced by ERG and not by the ERG-K362A, supporting the notion that impairing methylation reduced the tumorigenic ability of ERG and abolished the growth advantage given by WT-ERG (Fig. 3i and Supplementary Fig. 4g, h). Accordingly, immunostaining of the proliferation marker Ki67 was reduced significantly in ERG-K362A compared to WT-ERG xenografts (Fig. 3i left). Furthermore, the ERG DM promoted in vivo growth of RWPE1 xenografts significantly more than control vector (EV), K362A and WT-ERG, supporting the link between ERG conformational changes and enhanced oncogenic activity (Fig. 3j). This was also supported by the ability of the ERG DM to induce EZH2 expression in RWPE1 xenograft significantly more than WT and K362A mutant (Supplementary Fig. 4i). Taken together, these data support the notion that methylation promotes ERG activity, transcriptional and phenotypic reprogramming in prostate cancer cells. Collectively, both in vitro and in vivo data underline the relevance of K362 methylation and the consequent conformational changes as a molecular event enhancing ERG oncogenic activity.

### EZH2 enhances ERG transcriptional activity

**Fig. 4:**
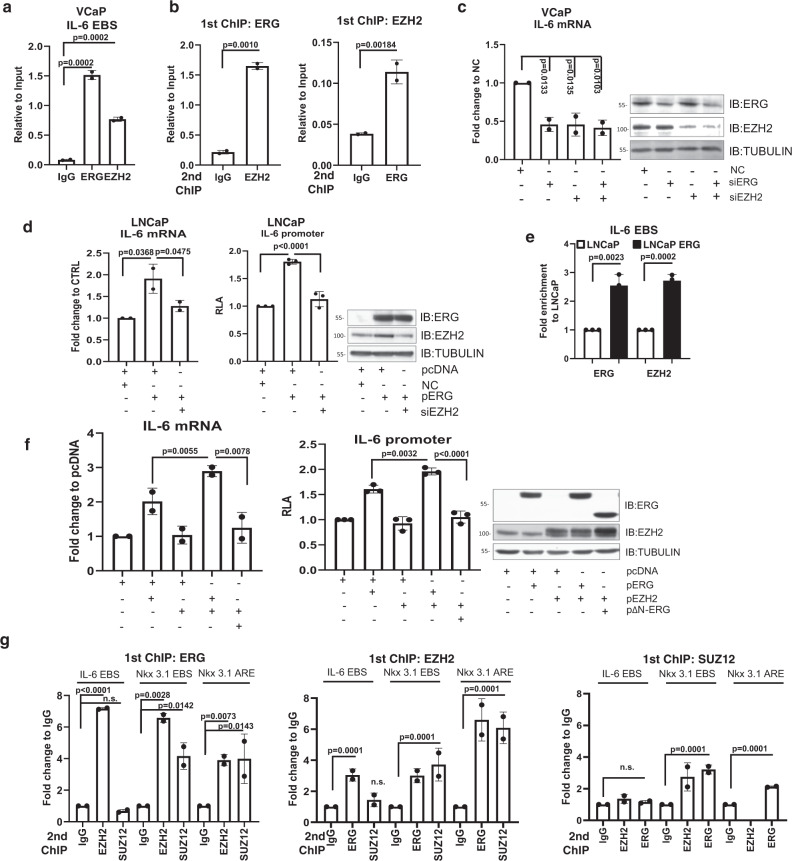
ERG and EZH2 co-occupy gene promoters forming activatory and repressory complexes. **a** ChIP analysis of ERG and EZH2 occupancy on the IL-6 promoter in VCaP cells. Data are presented as fold enrichment relative to input. **b** ChIP–reChIP analysis of ERG and EZH2 to evaluate co-occupancy at the IL-6 promoter in VCaP cells. Data are presented as fold enrichment relative to input. **c** qRT-PCR analysis of IL-6 mRNA and immunoblotting analysis of the indicated proteins (right) upon ERG and EZH2 knockdown in VCaP cells. **d** qRT-PCR analysis of IL-6 mRNA (left) and IL-6 promoter activity by luciferase reporter assay (middle) in LNCaP cells with ERG overexpression and EZH2 knockdown. Expression of the indicated proteins was verified by immunoblotting (right panel). **e** ChIP analysis of ERG and EZH2 occupancy in the IL-6 promoter at the ETS binding site (EBS) in LNCaP and LNCaP-ERG stable cell lines. **f** IL-6 mRNA determined by qRT-PCR (left) and IL-6 promoter activity by luciferase reporter assay (middle) in LNCaP transfected with the indicated plasmids. Expression of the indicated proteins was verified by immunoblotting (right). **g** ChIP–reChIP analysis of EZH2, ERG, and SUZ12 to evaluate co-occupancy at the IL-6 and Nkx3.1 promoters in VCaP cells. Data are presented as fold enrichment relative to IgG of the Re-ChIP. All error bars, mean ± s.d. (*n* = 3, technical replicates). *P*-values were determined by one-way ANOVA test. Source data are provided as a Source Data File.

Our data indicated that K362 methylation modulated ERG activity and this was a direct consequence of EZH2 interaction and ERG methylation. To assess the direct impact of EZH2 on ERG activity we performed multiple assays in different cell models. In RWPE1 cells expressing ERG, WT-EZH2, or catalytic inactive EZH2-ΔSET, luciferase reporter assays showed that co-expression of EZH2, but not EZH2-ΔSET, increased ERG-induced trans-activation (Supplementary Fig. 5a). Thus, EZH2 enhanced ERG function and this effect required EZH2 catalytic activity. In VCaP cells, using ChIP coupled with re-ChIP (ChIP–reChIP), we found that ERG and EZH2 bound at the IL-6 promoter, a known ETS-regulated target^19^ (Fig. 4a, b). Furthermore, individual or combined knockdown of ERG and EZH2 reduced IL-6 transcription (Fig. 4c). Thus, ERG and EZH2 interacted at the endogenous IL-6 promoter and together promoted its transcription. Consistently, expression of ERG in LNCaP cells induced IL-6 transcription and promoter activity and knockdown of EZH2 by siRNAs completely abolished this effect (Fig. 4d and Supplementary Fig. 5b). Interestingly, ectopic expression of ERG increased binding of both ERG and EZH2 to the IL-6 promoter, suggesting that ERG favored the recruitment of EZH2 to trans-activating complexes (Fig. 4e). In support of the relevance of the ERG/EZH2 interaction, the ΔN-ERG mutant, that could not bind EZH2, was unable to induce IL-6 transcription and promoter activity in LNCaP cells despite the overexpression of EZH2 (Fig. 4f). Thus, the ERG–EZH2 functional interaction involved both ERG methylation and formation of ERG/EZH2 complexes at the targeted promoters and was relevant for ERG trans-activating function.

Consistent with the canonical repressive function of EZH2, we found that EZH2 knockdown prevented ERG-induced repression of Nkx3.1 in ERG expressing LNCaP cells (Supplementary Fig. 5c). Furthermore, both ERG and EZH2 bound the Nkx3.1 promoter at nearby androgen receptor enhancer (ARE) (Supplementary Fig. 5d). This was associated with increased H3K27me3 at these sites in LNCaP-ERG cells compared to parental LNCaP cells (Supplementary Fig. 5e). Notably, we found that the presence or absence of SUZ12, a member of the PRC2 repressive complex^11^, could discriminate between activating and repressive complexes formed at the IL-6 and Nkx3.1 promoters. SUZ12 was present at the Nkx3.1 promoter and absent at the IL-6 promoter, despite similar promoter occupancy of the sites by ERG and EZH2 (Supplementary Fig. 5f). Furthermore, knockdown of SUZ12 prevented ERG-induced repression of Nkx3.1, but did not affect ERG-induced activation of IL-6 transcription (Supplementary Fig. 5g). Finally, in line with the formation of distinct regulatory complexes, ChIP–reChIP showed that neither ERG nor EZH2 interacted with SUZ12 at the IL-6 promoter, whereas SUZ12 co-localized with ERG and EZH2 at the Nkx3.1 promoter (Fig. 4g). Thus, depending on the promoter context, ERG and EZH2 formed non-canonical trans-activating complexes devoid of SUZ12 along with canonical repressive complexes containing SUZ12. Interestingly, despite the ability of SUZ12 and EED to bind ERG in whole cell lysates (Supplementary Fig. 5h), the formation of ERG/EZH2/SUZ12 complexes occurred exclusively at the repressed Nkx3.1 promoter.

### ERG/EZH2 co-occupancy occurs at multiple genomic sites and is frequently associated with transcriptional activation

**Fig. 5:**
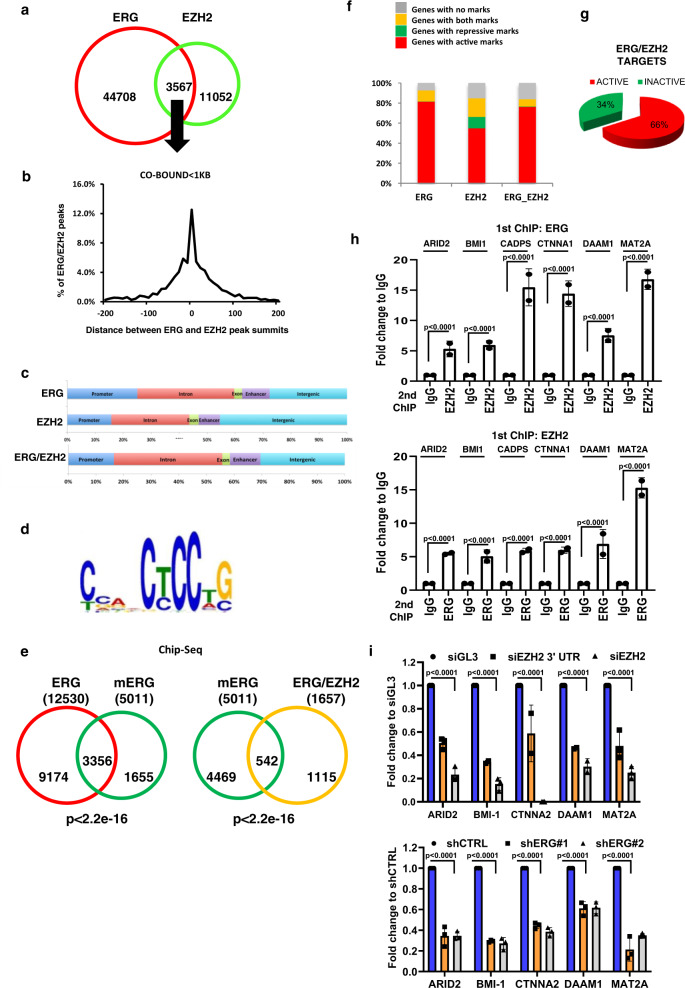
ERG and EZH2 co-occupy and co-regulate a specific network of genes. **a** Venn diagram showing the number of ERG, EZH2, and ERG/EZH2 co-localized peaks (≤1-kb) in VCaP cells extracted by ChIP-seq. **b** Distance of ERG and EZH2 peaks at ERG/EZH2 co-occupied sites. **c** Distribution of total ERG and EZH2 (top panel) and ERG–EZH2 co-occupied (lower panel) sites in intergenic, promoter, enhancer, intron, and exon regions. **d** Enrichment of ERG binding motif at ERG/EZH2 co-occupied sites by de-novo motif analysis. **e** Venn diagram showing the convergence of ERG and mERG genomic occupancy determined by ChIP-seq in VCaP cells. **f** Distribution of active and repressive histone marks among ERG, EZH2, and ERG/EZH2 targets. **g** Pie chart showing percentage of active and inactive genes among ERG/EZH2 targets in VCaP cells. **h** ChIP–reChIP analysis to evaluate co-occupancy by ERG and EZH2 at the indicated gene promoters in VCaP cells. *P*-values were determined by unpaired, two-tailed Student’s *t*-test. **i** Expression of ERG/EZH2 co-occupied genes after EZH2 (upper) or ERG (lower) knockdown in VCaP cells evaluated by qRT-PCR. All error bars, mean ± s.d. (*n* = 3, technical replicates). *P*-values were determined by one-way ANOVA test. Source data are provided as a Source Data File.

To understand broadly the functional impact of ERG/EZH2 co-occupancy at the genomic level, we examined available ERG and EZH2 ChIP-Seq data from VCaP cells^20^. We mapped 14,780 and 48,274 binding events for EZH2 and ERG, respectively (Fig. 5a). Co-localization of ERG and EZH2 (defined as peaks with summits within a ≤1-kb window) occurred at 3567 genomic sites, which represented 7.4 and 24.1% of the total ERG and EZH2 binding events, respectively. At these locations, we found a substantial overlap of the binding sites of ERG and EZH2 proteins with ≥70% of the sites having the respective peak summits within ±200 bp from each other (Fig. 5b). Indeed, ≥90% of the co-occupied sites had a distance between the peak summits within 20 bp from each other, confirming the interaction between the two proteins and consistent with the ChIP/re-ChIP data.

ERG, EZH2, and ERG/EZH2 co-occupied sites had similar distribution and were located in promoter, enhancer, intron, exon, and intergenic regions (Fig. 5c and Supplementary Fig. 6a). Interestingly, high-intensity ERG binding among the co-occupied sites prevailed at enhancers (Supplementary Fig. 6a). On average, ERG binding was higher at the co-occupied sites compared to ERG solo-sites, consistent with a positive effect of EZH2 on ERG binding (Supplementary Fig. 6a). De novo motif analysis revealed that EZH2 peaks in close proximity of ERG peaks (≤1-kb apart, *n* = 3442) were enriched of ERG binding motif (Fig. 5d), whereas the more distal EZH2 peaks (>1-kb window, *n* = 11,339) were not, in line with the experimental evidence that ERG guided recruitment of EZH2 to ERG target sites. Notably, ChIP-Seq analysis with the anti-mERG antibody in VCaP cells showed a significant overlap between mERG and ERG-bound genes with about 70% of the mERG overlapping with ERG (Fig. 5e). There was also a significant overlap between mERG-bound genes and ERG/EZH2 co-occupied genes (Fig. 5e) sustaining the functional link between, ERG and EZH2 interaction, ERG methylation and genomic occupancy.

Notably, analysis of epigenetics marks in VCaP cells^5,21^ indicated that the ERG/EZH2 co-occupied genes were predominantly associated with activating histone modifications similar to ERG-bound genes and in line with active transcription (Fig. 5f). Integrating ChIP-Seq and RNA-Seq data from VCaP cells^22^, we found that the majority of ERG/EZH2 (66%) co-occupied genes were transcriptionally active (Fig. 5g). To validate these findings, we focused on a set of 5 genes with strong and overlapping ERG/EZH2 predicted peaks and activating histone marks in VCaP cells (Supplementary Fig. 6b). Moreover, the scores for these 5 genes for both ERG and EZH2 were top ranking among the all list of co-occurring peaks (Supplementary Fig. 6b). Consistently, analysis by ChIP–reChIP revealed concomitant occupancy of ERG and EZH2 at all selected peak sites (Fig. 5h). Consistent with trans-activation of these targets by ERG and EZH2, gene expression was reduced significantly upon RNAi-induced knockdown of ERG and EZH2 (Fig. 5i and Supplementary Fig. 6c). Notably, the expression level and promoter occupancy of several ERG/EZH2 co-bound targets was significantly higher in WT-ERG compared to ERG-K362A mutant expressing LNCaP cells, supporting the role of K362 methylation in enhancing ERG transcriptional activity (Supplementary Fig. 6d–e).

### ERG/EZH2 activation is associated with PTEN deletion and tumor progression in transgenic/knockout mice

**Fig. 6:**
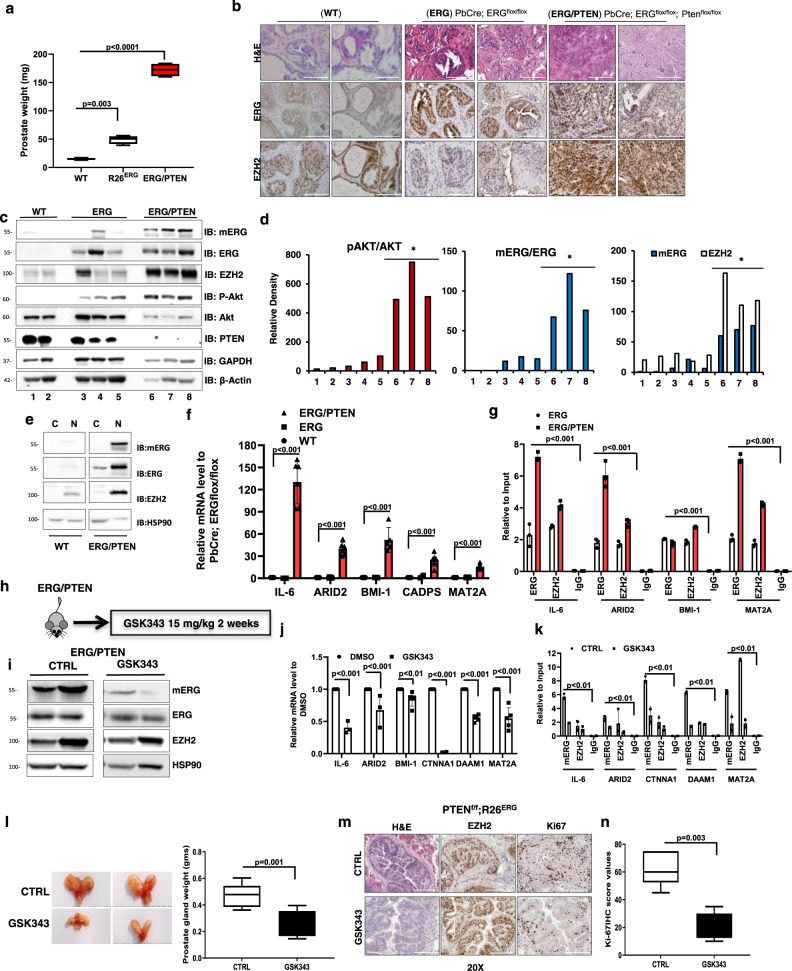
ERG/EZH2 activation is associated with PTEN deletion and tumor progression in transgenic/knockout mice. **a** Box-plots of prostate weights evaluated at 16-week from wild-type (ERG flox/flox) ERG (PbCre; ERG flox/flox) and ERG/PTEN (PbCre; ERG flox/flox PTEN flox/flox) mice (*n* = 4/group). Minima in WT group is 13, maxima is 17, median is 15, percentile (0,25^th^) is 13.25; minima in R26^ERG^ group is 39.2, maxima is 56.1, median is 49.05, percentile (0,25^th^) is 41.57; minima in ERG/PTEN group is 165.2, maxima is 183.6, median is 172.2, percentile (0,25^th^) is 161.3. **b** Histological and IHC evaluation from 16-week old prostate from wild-type, ERG, and ERG/PTEN mice. FFPE representative prostate sections were examined by IHC to assess ERG and EZH2 (*n* = 3). Scale bars represent 200 µm. **c** Prostates of 16-week-old wild-type, ERG, and ERG/PTEN mice (*n* = 3) were examined by immunoblots to assess mERG, ERG, and EZH2. Lysate were collected at 16 weeks from ventral prostate lobes and analyzed with indicated antibodies. **d** Densitometric analysis of pAKT relative total AKT (left panel), mERG relative to ERG (middle panel) mERG and EZH2 relative to GAPDH and (right panel). **e** Cytoplasmic and nuclear localization of ERG, EZH2, and mERG in prostate tissues from WT and ERG/PTEN mice (*n* = 2). **f** Expression of selected ERG/EZH2 target genes evaluated by qRT-PCR in prostatic tissue from 16-week-old ERG (PbCre; ERG flox/flox) and ERG/PTEN (PbCre; ERG flox/flox PTEN flox/flox) mice. (*n* = 3/ biological independent samples). **g** ChIP-qPCR analysis of ERG and EZH2 occupancy at the indicated promoters in prostatic tissue from 16-week-old ERG (PbCre; ERG flox/flox) and ERG/PTEN (PbCre; ERG flox/flox PTEN flox/flox) mice. (*n* = 3/biological independent samples). **h** Experimental plan. 25-week-old ERG/PTEN mice (*n* = 5/group) were treated with vehicle or 15 mg/kg GSK343 by intraperitoneal injection three times/week for 2 weeks. **i** Immunoblotting analysis of the indicated proteins in prostatic tissue from control and GSK343-treated ERG/PTEN mice (*n* = 2). **j** Expression of ERG/EZH2 co-regulated genes in control and GSK343-treated ERG/PTEN mice evaluated by qRT-PCR. **k** ChIP-qPCR analysis of mERG and EZH2 occupancy at the indicated promoters in prostatic tissue from control and GSK343-treated ERG/PTEN mice. **l** Prostate tumor development in ERG/PTEN mice treated with vehicle (control) or GSK343. 25-week-old ERG/PTEN mice (*n* = 5/group) were treated with vehicle or 15 mg/kg GSK343 by intraperitoneal injection three times/week for 2 weeks. Left, representative images of prostates. Right, prostate weights at the end of the experiment. Minima in CTRL group is 0.362, maxima 0.6031, median is 0.467, percentile (0,25^th^) is 0.385; minima in GSK-343 group is 0.145, maxima 0.39, median is 0.263, percentile (0,25^th^) is 0.16. **m** H&E staining and IHC evaluation of ERG, EZH2, and Ki67 in ERG/PTEN mice following treatment with vehicle or GSK343 as described above (*n* = 2). Scale bars represent 200 µm. **n** Quantitative IHC scores of Ki67 immunostaining in control and GSK343-treated ERG/PTEN mice. *P*-values were determined by one-way ANOVA test. Minima in CTRL group is 45, maxima 75, median is 63, percentile (0,25^th^) is 52.5; minima in GSK-343 group is 10, maxima 35, median is 20, percentile (0,25^th^) is 12.5. All error bars, mean ± s.d. (*n* = 3, technical replicates). Source data are provided as a Source Data File.

To gain evidence of the link between ERG methylation and prostate cancer progression, we took advantage of the Pb-Cre4;Rosa26^*ERG/ERG*^ (ERG mice) with prostate-specific expression of ERG and of the combined Pb-Cre4;Pten^flox/flox^;Rosa26^*ERG/ERG*^ (ERG/PTEN mice) with prostate-specific ERG expression and PTEN deletion. These extensively characterized mice represent a model of ERG positive prostate tumor progression from an indolent to an aggressive stage^8^. ERG transgenic mice fail to develop invasive lesions, whereas the combined ERG/PTEN mice develop invasive prostate adenocarcinomas^8^. At 16 weeks of age we observed increased prostate size (Fig. 6a) and invasive lesions at the histopathological analysis (Fig. 6b) in ERG/PTEN mice and not in WT and ERG mice. ERG was expressed in prostatic tissue from ERG and ERG/PTEN mice as shown by immunohistochemistry and immunoblotting (Fig. 6b, c). Immunoblotting confirmed loss of wild-type PTEN and enhanced serine 473 AKT phosphorylation (pS473 AKT) in the ERG/PTEN mice compared to ERG and WT mice (Fig. 6c, d). In line with our hypothesis, mERG was not detected in WT and barely detectable in ERG mice and increased substantially in ERG/PTEN mice both in total amount than in relation to ERG (Fig. 6c, d). EZH2 level was also higher in ERG/PTEN compared to WT and ERG mice (Fig. 6b–d). Furthermore, as seen in VCaP cells (Fig. 1d), mERG was predominantly nuclear in the prostates of ERG/PTEN mice, similar to ERG and EZH2 (Fig. 6e). In line with enhanced ERG activity by K362 methylation, the expression of co-bound ERG/EZH2 target genes and promoter occupancy by ERG was increased in ERG/PTEN mice compared to WT or ERG mice (Fig. 6f, g). Systemic treatment of ERG/PTEN mice with the EZH2 inhibitor GSK343, reduced ERG methylation (Fig. 6h, i). Notably, this resulted in significantly reduced expression of ERG/EZH2 target genes and ERG occupancy on their promoters (Fig. 6j, k). Moreover, concomitantly with the ERG transcriptional reversion, treatment with GSK343 caused in ERG/PTEN mice a striking reduction of the tumor burden, estimated as total prostate volume, compared to vehicle-treated control mice (Fig. 6l). This was also associated with a reduction of invasive areas at the histopathological examination (Fig. 6m) and tumor proliferative activity determined by Ki67 immunostaining (Fig. 6n). Collectively, these data demonstrates that inhibition of EZH2 reverse both ERG transcriptional and oncogenic activation.Thus, these data strongly support a link between ERG methylation by EZH2 and enhanced ERG transcriptional activity in ERG/PTEN mice.

**Fig. 7:**
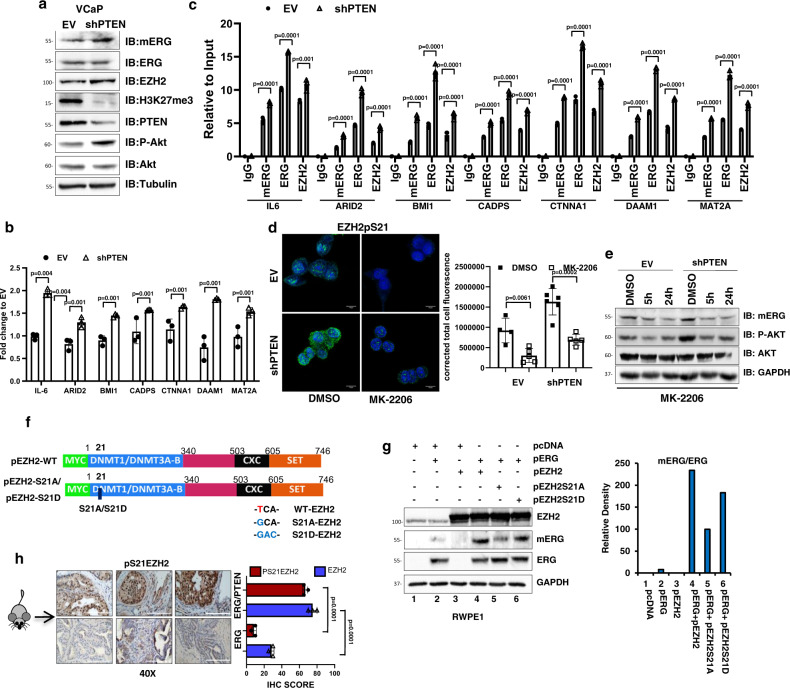
PTEN deficiency enhances ERG methylation and transcriptional activity. **a** Immunoblots of mERG and the indicated proteins in control (EV) and stable PTEN knockdown (shPTEN) VCaP cells (*n* = 3). **b** Expression of ERG/EZH2 co-occupied genes in VCaP-shPTEN and control (EV) VCaP cells evaluated by qRT-PCR. **c** ERG, mERG, and EZH2 occupancy at the indicated gene promoters in VCaP-shPTEN and control (EV) VCaP cells by ChIP-qPCR. **d** EZH2 phosphorylation (Ps21) evaluated by IF and quantitative assessment (right) in VCaP-shPTEN and control (EV) following treatment with MK-2206 (5 µM) for 24 h. Scale bar 10 µm (*n* = 2). **e** Immunoblots of mERG and indicated proteins in VCaP-shPTEN (sh-PTEN) and control (EV) cells treated with DMSO and MK-2206 (5 µM) for 5 and 24 h (*n* = 2). **f** Diagram of WT, S21A, and S21D EZH2 mutated constructs. **g** Immunoblots of mERG and indicated proteins in RWPE1 cells transfected with indicated expression plasmids. Right, densitometric assessment of mERG level relative to total ERG (*n* = 2). **h** IHC evaluation of pS21 EZH2 in prostate tissue from ERG (PbCre; ERG flox/flox) and ERG/PTEN (PbCre; ERG flox/flox PTEN flox/flox) mice. Right, quantitative IHC scores of pS21EZH2 and EZH2. All error bars, mean ± s.d. (*n* = 3, technical replicates) (**b**, **c**). *P*-values were determined by one-way ANOVA test. Scale bars represent 200 µm. Source data are provided as a Source Data File.

To gain further mechanistic insights on the signaling pathways promoting ERG methylation in the context of PTEN loss, we knocked down PTEN in ERG-positive PTEN wild-type VCaP cells. In line with the findings in the mouse model, knockdown of PTEN in VCaP cells increased pS473 AKT (Fig. 7a). Importantly, PTEN knockdown enhanced ERG methylation and increased expression level and promoter occupancy by ERG, mERG, and EZH2 of multiple ERG/EZH2 co-regulated targets (Fig. 7a–c). Moreover, PTEN-depleted VCaP cells exhibited enhanced ability to invade blood vessels and form liver metastases in CAM assays (Suplementary Fig. 7d–e). Notably, the EZH2 inhibitor GSK343, reversed both transcriptional and functional effects of PTEN knockdown, in line with their association with EZH2-induced ERG methylation and activation (Supplementary Fig. 7a–g). These data support a strict link between PTEN loss and EZH2-induced ERG transcriptional activity.

Collectively, these data suggested a strong link between PTEN deficiency and ERG activation by EZH2. Mechanistically, we hypothesized that PTEN loss and AKT activation could lead to phosphorylation of EZH2 at serine 21 (pS21), which could favor methylation of non-histone proteins^23^. Consistently, we found increased pS21 EZH2 in VCaP-shPTEN cells compared to control cells, which was significantly reduced by the AKT inhibitor MK-2206 in both control and shPTEN cells (Fig. 7d). Moreover, AKT inhibition by MK-2206 reduced mERG in these cells (Fig. 7e), linking AKT, with EZH2-induced ERG methylation. In support of the relevance of S21 EZH2 phosphorylation, we generated a phosphorylation-defective EZH2-S21A mutant (EZH2-S21A) (Fig. 7f) and determined its ability to induce ERG methylation in comparison with wild-type EZH2 (WT-EZH2) upon the expression in RWPE1 cells (Fig. 7g). The EZH2-S21A had reduced ability to induce methylation of ERG compared to WT-EZH2 and the phosphorylation-mimic EZH2-S21D in RWPE1 cells (Fig. 7g). Together, these data support the notion that PTEN loss and AKT activation, through the induction of pS21 EZH2 phosphorylation, promote ERG methylation. Notably, pS21 EZH2 phosphorylation and EZH2 level were significantly increased also in ERG/PTEN mice compared to ERG mice (Fig. 7h). Notably, in ERG/PTEN mice the percentage of pS21 EZH2 phosphorylated compared to total EZH2 was significantly higher than in ERG mice (89 versus 29%, respectively). These data were also consistent with the increased expression level of selected ERG/EZH2 targets and promoter occupancy by ERG observed in ERG/PTEN compared to WT and ERG mice (Fig. 6f, g).

### ERG/EZH2 co-regulated genes are associated with aggressive disease

**Fig. 8:**
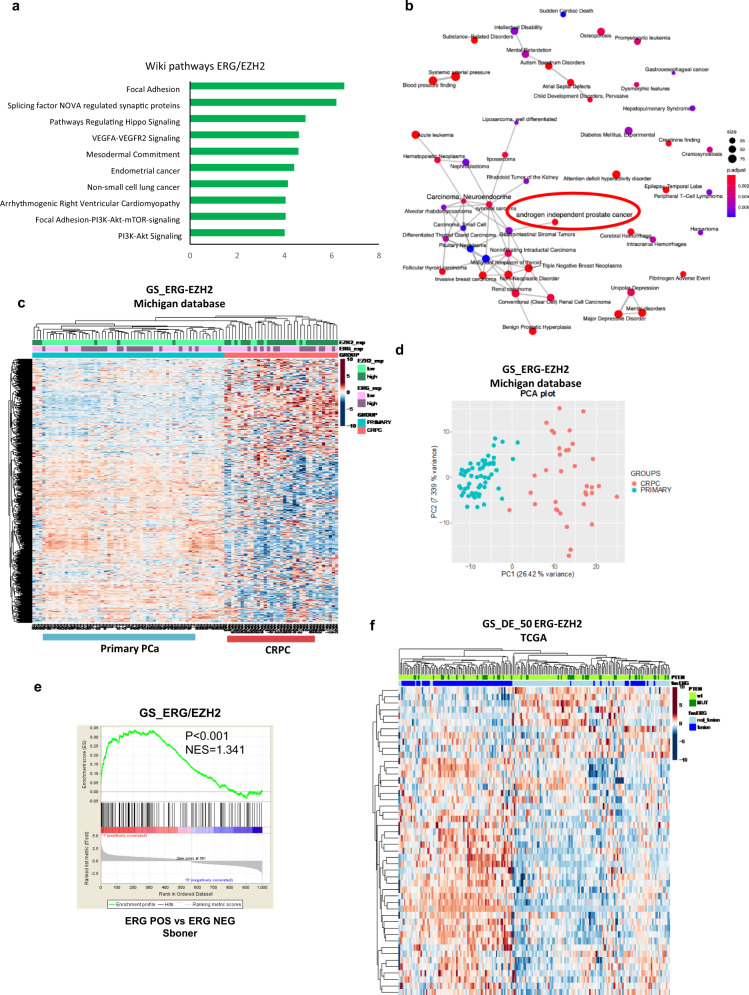
ERG/EZH2 co-regulated genes are expressed in aggressive prostate tumors. **a** Pathways enriched in the list of genes co-occupied by ERG/EZH2 (GS_ERG/EZH2, *n* = 1656 genes) using enrich tool. **b** Functional analysis of the GS_ERG/EZH2 set using enrichDGN tool. **c**, **d** Heat map (**c**) and principal component analysis (**d**) using the GS_ERG/EZH2 set applied to primary prostate tumors (PRIMARY) and castration-resistant prostate cancers (CRPC) with known ERG and EZH2 status in the Michigan dataset. Color bars indicate ERG and EZH2 expression status. **e** GSEA using the GS_ERG/EZH2 set comparing ERG fusion positive and negative prostate tumors in the Sboner dataset. **f** Heat map with the top deregulated ERG/EZH2 gene signature (GS_ERG/EZH2_50) applied to the TCGA dataset. Color bars indicate ERG fusion and PTEN status.

Our data in cell lines and mouse models indicate that methylation and enhanced trans-activation capability of ERG would result in activation of ERG/EZH2 co-regulated genes relevant for disease progression. Interestingly, the co-occupied ERG/EZH2 target genes in VCaP cells (*n* = 1656) were functionally associated with pathways, such as focal adhesion and adherent junctions, related to cell migration and invasion and other features indicative of dedifferentiation and stemness in prostate epithelial cells (i.e., neuronal axon, Hyppo signaling) (Fig. 8a). Interestingly, exploration of human gene disease network using *DSgnet* tools revealed also a relation with features altered in several cancers, including androgen-independent prostate cancer (Fig. 8b, *p* < 0.0001). Consistently, the mERG/EZH2 gene set (*n* = 540), was functionally associated with oncogenic pathways and features enriched in cancer most of them overlapping with the ERG/EZH2 gene set (Supplementary Fig. 8a, b).

To further understand the relevance of these findings in human tumors, we examined the expression of the ERG/EZH2 target genes in transcriptomic data from prostate cancer patients. In line with a link with disease progression, the ERG/EZH2 co-regulated gene set (GS_ERG/EZH2), separated clearly CRPCs from primary tumors in unsupervised clustering analysis and were significantly deregulated in CRPCs compared to primary tumors (Fig. 8c). Notably, concomitant expression of ERG and EZH2 was more frequent in CRPCs than primary tumors, justifying the prominent deregulated expression of their targets. A clear separation was also appreciated by principal component analysis (Fig. 8d). Intriguingly, the genes deregulated in CRPC were both enhanced (44%) and repressed (56%) supporting that ERG methylation favor ERG transcriptional activity in both directions (Supplementary dataset 2). Consistently, similar clusterization was observed for the mERG/EZH2 targets with both significantly enhanced (50%) and attenuated (50%) genes in CRPC versus primary prostate tumors (Supplementary Fig. 8c and Supplementary dataset 3).

We found increased ERG methylation and activation in association with PTEN loss in both cellular and mouse models of ERG fusion positive tumors. In support of this, we observed significant enrichment (*P* < 0.001) of ERG/EZH2 co-regulated genes by Gene Set Enrichment Analysis (GSEA) in ERG fusion positive compared to ERG fusion negative primary tumors in the Sboner dataset^24^ (Fig. 8e).

Next, we extracted a list of the most significantly deregulated ERG/EZH2 targets between the two tumor subgroups (ERG fusion positive vs ERG fusion negative tumors) in the Sboner dataset^24^ (Supplementary Fig. 9). We reasoned that these genes might closely mirror the degree of ERG/EZH2 activation in prostate cancers. Interestingly, when applied to primary prostate tumors in the TCGA dataset, these genes clearly discriminated ERG-positive tumors with PTEN deficiency (by deletion or mutation) from wild-type tumors for both ERG fusion and PTEN loss (Fig. 8f). Thus, activation of the ERG/EZH2 co-regulated genes is frequently concomitant with ERG fusion and PTEN loss also in human tumors and is more prominent in CRPCs. These data are relevant for primary prostate cancer stratification and point to a determining role of EZH2-mediated ERG activation in the acquisition of aggressive features and poor prognosis.

## Discussion

Aberrant expression of the transcription factor ERG produces profound changes in the transcriptome of prostate epithelial cells. However, the mechanisms underlying ERG-induced tumorigenesis and its impact on tumor progession are still unclear. In this study, we uncovered a relevant mechanism promoting progression in ERG-fusion positive prostate cancer. We found that EZH2 interacts with ERG and catalyzes mono-methylation of K362 in ERG. Both physical interaction and K362 methylation are instrumental to induce the broad transcriptional and phenotypic reprogramming driven by ERG in prostate tumors. Through this physical and functional interaction, ERG and EZH2 cooperatively activate a network of genes sustaining tumor progression and castration resistance.

Lysine methylation of non-histone proteins has emerged as an important post-translation modification that affects protein function^25,26^. EZH2 has been reported to methylate a number of non-histone proteins^14,15,27,28^. The functional consequences of the methylation may vary depending on the substrate protein and include changes in protein degradation, protein–protein interaction, intracellular localization, and catalysis^25,26,29^. In this study, we report an important mechanism involving conformational changes induced by lysine methylation that affect DNA binding and trans-activating capability of ERG. We found that mono-methylation of K362 changes the intra-molecular dynamic interactions within the internal auto-inhibitory modules and their relation with the DNA binding and trans-activating domain. In the auto-inhibited state, the central DNA-binding module (α3 helix) is trapped in a hydrophobic cage formed by the NID and the α4 helix of the CID^18^. Consistently, deletions of the NID and CID modules enhance DNA binding and transcriptional activity^18^. The K362 resides at the edge of the α2 helix in the short loop connecting α2 to the α3 helix and makes contacts with multiple amino acids, including E412 in the α4 helix. We proposed that the inter-molecular bridge between K362 and E412 could reduce the mobility and flexibility of the CID, thereby locking ERG in the auto-inhibited state. K362 methylation disrupts the K362–E412 interaction, thus favoring the release of the α3 helix and facilitating DNA binding.

Further evidence of a major conformational switch associated with K362 methylation came from comparing in silico the structure of uninhibited DNA-bound ERG and those of the auto-inhibited, methylated, and mutated forms. Importantly, the K362-methylated ERG assumed a conformation similar to that of the uninhibited DNA-bound ERG. Conversely, the K362A mutant retained a conformation closer to the auto-inhibited ERG. Surprisingly, we found mutating both K362 and E412 and completely disrupting any possible interaction between the two residues, induced a conformation very stable and similar to that of uninhibited ERG domain and thus highly favorable for DNA binding. Functional assays showed that preventing methylation with the K362A mutation reduced ERG-induced transcriptional response along with tumorigenic phenotypes in both cell cultures and tumor xenografts. Conversely, consistent with the MDS predictions, the double-mutant K362A/E412A enhanced transcription in reporter assays, chromatin binding at endogenous promoters, and, in RPWE1 cells, in vivo tumor growth.

The network of co-regulatory factors recruited by ERG has a paramount role in prostate tumorigenesis. ERG together with co-repressors, like HDACs and EZH2, modulates AR transcriptional activity, impeding epithelial differentiation and contributing to tumor progression^20^. ERG disrupts AR signaling and potentiate EZH2-mediated epigenetic repression of multiple targets^5^. On the other hand, Kim et al. have shown recently that EZH2 can act also as transcriptional activator with respect to AR and AR-regulated genes, a function apparently independent of the PCR2-mediated gene silencing^30^. Our study reveals an additional mechanism that enhances ERG-induced reprogramming of the prostate cancer cell transcriptome. Indeed, our data reveal a completely new aspect of the ERG/EZH2 partnership. Underlying the functional relevance of this interaction, ERG-induced transcriptional activity was blocked by depliting or inhibiting EZH2. Mechanistically, we found that ERG and EZH2 can form transcriptional activating complexes and that EZH2 sustains ERG trans-activation. Thus, in this context, EZH2 acts as ERG co-activator in a non-canonical PRC2-independent mode. These findings are in line with emerging PRC2-independent functions of EZH2^12,31^. However, we provide additional insights showing that the non-canonical function of EZH2 in ERG-positive prostate tumors involves the direct interaction, methylation, and functional cooperation with ERG resulting in enhanced trans-activation of selected co-regulated target genes.

Analysis of ChIP-seq data from VCaP cells supports our conclusions showing that ERG and EZH2 co-occupy several sites in the human genome. Motif analysis confirmed the presence of a canonical ERG motif at EZH2 peaks proximal to ERG-binding sites suggesting that EZH2, driven by ERG, gains additional binding sites in the genome. ERG–EZH2 co-occupied sites corresponded prevalently to transcriptionally active genes. Furthermore, genomic occupancy of mERG coincided largely with ERG and ERG/EZH2 targets in VCaP cells, thus linking ERG methylation and enhanced ERG trans-activation. In support of their relevance to progression, we found that ERG/EZH2 coregulated targets were significantly deregulated in CRPC compared to primary prostate tumors. Intriguingly, the coregulated targets were both enhanced and repressed supporting the notion that ERG methylation favors ERG transcriptional activity in both directions.

An event that cooperates with ERG upregulation in prostate cancer progression is loss of PTEN. PTEN deficiency and AKT activation have been proposed as “second hit” that makes oncogenic function of ERG more penetrant^9,10^. We found increased ERG methylation in ERG/PTEN mice compared to ERG and WT mice. Thus, enhanced ERG methylation could provide a mechanistic explanation for the cooperative effect of ERG gain and PTEN loss. As supporting evidence, we found that PTEN knockdown in VCaP cells increased mERG and AKT inhibition reduced mERG. PTEN depletion in VCaP cells led to further activation of AKT and induction of pS21 EZH2. Specifically, AKT-induced pS21 phosphorylation was proposed to suppress canonical H3K27 methylation and switch EZH2 towards non-histone substrates^23^. Consistently, PTEN-depleted VCaP cells exhibited reduced global H3K27 methylation and increased mERG, in line with a switch in substrate preference. pS21 phosphorylation of EZH2 has also been proposed to promote non-canonical EZH2 functions and switch from co-repressor to co-activator functions. Our data also support this hypothesis showing that PTEN depletion, likely through ERG methylation and EZH2 interaction, enhanced trans-activation by ERG/EZH2 complexes in ERG-positive cancer cells. Furthermore, ERG–EZH2 genomic co-occupancy and expression of several co-regulated genes increased concomitant to PTEN loss in cell lines, mouse models, and patient samples, confirming the link between PTEN loss and enhanced ERG activity. A limitation of our study is that we were able to evaluate mERG expression by immunoblotting only in a small set of prostate tumors, from which we had sufficient amounts of tissue. These data support the presence of mERG in ERG fusion positive human cancers. However, due to poor performance of the anti-mERG antibody in immunohistochemistry, we could not assess presently larger numbers of tumor tissue specimens to study the frequency and association of mERG expression with clinical parameters and outcome.

In summary, this study provides insights into the epigenetic reprogramming caused by ERG in prostate cancers and shows the key role of ERG–EZH2 functional interaction in promoting tumor progression. We propose ERG methylation as a critical post-translational modification enabling the activation of pro-tumorigenic and pro-metastatic programs. These findings also define a therapeutically actionable pathway, modulation of which might have an important impact in the treatment of ERG fusion positive prostate cancer. Multiple mechanisms may trigger ERG methylation and discovering them could be a significant priority in the near future leading to therapeutic strategies to reverse this post-translational modification and disrupt the ERG co-regulatory network responsible for tumor progression.

## Methods

### Cell cultures

VCaP, LNCaP, and PC3 were obtained from ATCC and maintained in DMEM (VCaP) or RPMI-1640 (Gibco) supplemented with 10% fetal bovine serum. All cell lines were authenticated by DNA profiling (short tandem repeat analysis) and were used within 6 months of culturing. RWPE1 cells were maintained in keratinocyte serum-free growth medium (Gibco) with specific supplements^32^. Cells were regularly checked for mycoplasm contamination using MycoAlert Mycoplasma detection kit (Lonza). When indicated, cells were treated with DZNep (Cayman Chemical), GSK343 (Selleckchem), or MK-2206 (Selleckchem).

### Transfection, vectors, and generation of stable cell lines

For transient gene knock-down, cells were transfected with siRNAs directed to ERG (Qiagen), EZH2 (Ambion-Life technologies), or a control siRNA (siGL3) directed to the firefly luciferase gene (Ambion-Life technologies). Cells were transfected with 100 nM of siRNA using Lipofectamine 2000 (Invitrogen) and were harvested after 48 h. siRNAs and shRNAs are shown in Supplemenary Table 1, (see Supplementary info). PC3 and RWPE1 cells were transiently transfected with different ERG and EZH2 expression constructs using JetPRIME. The following constructs were used: pCEFL-ha-ERG (kindly provided by S. Izraeli), pcDNA3-myc-EZH2 (kindly provided by Jer-Tsong Hsieh), and pCDNA3-myc-EZH2-ΔSET (kindly provided by TaI-Lung Cha). ERG mutated or truncated constructs (i.e., ha-ΔC-ERG, ha-P-I-ERG, ha-P-ERG, ha-N-ERG, his-∆N-ERG, ERG-K362A, ERG-E412A, ERG-K362A_E412A) and EZH2 mutated or truncated constructs (i.e., EZH2-∆CXC, EZH2-1-340, S21A, and S1D EZH2) were generated using Quick Change Site-Directed Mutagenesis Kit (Stratagene) using pCEFL-ha-ERG and pCDNA3-myc-EZH2 vectors as templates, respectively. LNCaP with stable expression of wild-type ERG (ERG-WT), ERG mutant (ERG-K362A), or empty vector (EV) was generated by transfection of the corresponding expression vectors (pCEFL-ha-ERG, pCEFL-ha-ERG-K362A, pcDNA3.1) using JetPRIME (Polyplus) and selected with G418. ERG, ERG-K362A expression was determined by qRT-PCR and Western blotting. VCaP with stable PTEN depletion were generated by viral infection of control (EV) and PTEN shRNAs (shPTEN) (Sigma) and subsequent selection with puromycin. PTEN downregulation was evaluated by qRT-PCR and Western blotting.

### Custom-made anti-methyl-ERG antibody

The anti-methyl-ERG antibody (affinity purified rabbit polyclonal, AbMart) was custom-made and generated using a lysine (K362) mono-methylated ERG peptide. Specificity of the antibody was determined by peptide competition assay, in which the antibody was pre-incubated with the methylated and non-methylated competitor peptides prior to immunoblotting. Methyl-ERG antibody was pre-incubated with or without a 5-fold excess of competitor peptides 1 h at room temperature. Three identical samples of VCaP cells lysate were run on a gel and immunoblotted with the different antibody–peptide mixtures. The signals obtained with the antibody alone or antibody + non-methylated peptide were completely eliminated with the neutralized antibody (antibody + methylated peptide). Similar tests of specificity were performed for immunofluorescence microscopy detection.

### Immunoblotting and immunoprecipitation

Cell lysates were prepared lysing cells in RIPA lysis buffer (50 mM Tris-HCl pH 7.4; 150 mM NaCl; 1 mM EDTA; 1% NP-40; 0.25% Na-deoxycholate; 4 mM Na_3_VO_4_; 1 mM PMSF; 1 mM NaF) with protease inhibitor cocktail (Roche) and phosphatase inhibitor cocktail (PhosStop, Roche). Proteins were resolved on 10, 12, or 15% SDS PAGE and analyzed by Western blot. The following antibodies were employed: anti mERG (custom made 1:300), anti-ERG (sc-353 Santa Cruz Biotechnology Inc., 1:3000), anti-ERG rabbit monoclonal (Epitomics, 1:3000), anti-EZH2 (BD Biosciences, 1:3000), anti-PTEN (#9552 Cell Signaling, 1:2000), anti-pAkt (Ser473 #4051 Cell Signaling, 1:1000), anti-Akt (#9272 Cell Signaling, 1:2000), anti-AR (#06-680, Millipore, 1:1000), anti-HA (F7, Santa Cruz, 1:1000), anti-Histidine (H1029, Sigma Aldrich, 1:3000), anti-cMyc (BD Biosciences, 1:1000), anti-pan-methylated Lysine (ab23366 Abcam, 1:500), anti-Tri-Methyl-Histone H3 (Lys27) (C36B11, Cell Signaling, 1:2000), anti-EED (09-774, EMD Millipore, 1:2000), anti-SUZ12 (Ab12073, AbCam, 1:2000), anti-GAPDH (Santa Cruz, 1:10000), anti-α-tubulin (Calbiochem, 1:10000), anti-β-Actin (sc-1616, Santa Cruz Biotechnology Inc., 1:1000), anti-HSP90 (C45G5, Cell Signaling, 1:10000), and anti-GRP94 (#2104, Cell signaling, 1:1000). Quantification of the bands was performed using FusionCapt Advanced Solo7. Immunoprecipitation experiments were performed using Protein G PLUS/Protein A-Agarose mixture (Calbiochem-Millipore), which were incubated with 100–300 ug of lysates and 1–5 ug of anti-HA, anti-ERG (sc-354X, Santa Cruz Biotechnology Inc.), anti-ERG (Epitomics), anti-AR (#06-680, Millipore), anti-EZH2 (AC22, Active Motif), anti-EED (09-774, EMD Millipore), and anti-SUZ12 (Ab12073, AbCam) antibodies. Histidine pull-down was performed using Dynabeads His-Tag Isolation & Pulldown magnetic beads (Invitrogen).

### Immunofluorescence microscopy

To perform immunofluorescence cells were grown on glass cover-slips, fixed with 4% formaldehyde, permeabilized with 1:1 methanol–acetone, incubated with anti-ERG (Epitomics), anti-EZH2 (BD Biosciences), and anti-mERG as primary antibodies and then with anti-rabbit Alexa 594 or anti-mouse Alexa 488 (Invitrogen). To perform immunofluorescence for anti-pS21 EZH2 (Bethyl Laboratories) cells were grown in μ-slide (chambered coverslip) with 8 wells (ibidi). At a later stage, cells were fixed and permeabilized with methanol–acetone (1:1) solution. After cells blocked for 10 min with blocking buffer (Dako, Protein Block Serum- Free, Ref. X0909) and incubated for 1 h at room temperature with anti-pS21 EZH2 antibody (Bethyl Laboratories). Then incubation with a secondary goat anti-rabbit Alexa Fluor 488 IgG (Invitrogen) followed. Antibodies were diluted in Antibody Diluent (Boster, AR1016). Nuclear visualization was carried out with Hoechst Staining (Hoechst 33342, Thermo Scientific). Images were obtained using the confocal microscope.

### Enzyme-linked immunosorbent assay (ELISA)

ELISA high-binding 96-well plates (Corning) were coated using a solution 10 μg ml^−1^ of avidin dissolved in PBS by overnight incubation at 4 °C. Subsequently, PBS-1% BSA was used as blocking solution and the plates coated with the biotinylated peptides 10 μg ml^−1^.The peptides were prepared by Genscript with biotin attached to the C-terminus. The antibody was prepared by serial dilution (1:1) starting from a concentration equal at 1/60 of the pure antibody preparation. The titrated antibody was incubated with the peptides then, after washing, 1/2500 alkaline phosphatase (AP)-conjugated Goat Anti-Rabbit IgG (Jackson Immuno, AB_2337956) was added. After washing, para-nitrophenyl phosphate (p-NPP), (Sigma) was added and plates were read at 405 nm. Nonspecific binding to plates coated with a random control peptide was tested to exclude polyreactivity of the antibody.

### Dot-blot

ERG peptides, including non-methylated, mono-, di-, and tri-methylated peptide, were resuspended in PBS (1 mg/ml) and spotted on nitrocellulose membrane. Membrane was blocked in milk 10% for 10 min, washed with TBS-T, and incubated with primary mERG antibody (1:300 dilution) at 4 °C. After secondary Ab incubation the signal was developed using the Western Bright ECL (Witec) and acquired with the Fusion Solo S System. A positive signal was appreciated only with the ERG monomethylated peptide. These data demonstrate that the Ab recognize ERG mono-methylation. The Ab recognize the peptide only when mono-methylated. There is no signal with a di-tri and non-methylated peptide.

### In vitro methyltransferase assay

An in vitro methyltransferase assay was done using a nonradioactive colorimetric assay kit (Cayman Chemical) with a slight modification as suggested by the supplier. The methyltransferase reaction was first carried out in a 115-μL reaction mixture containing 500 ng of ERG recombinant protein (Origene, TP308093), 500 ng of recombinant PRC2 complex (Active Motif), 100 nM GSK343, and 100 μL SAM Master Mixture (Assay Buffer, MT Enzyme Mixture, MT Colorimetric Mixture and MT SAM). The samples were incubated for 30 min at 37 °C. The reactions were stopped by boiling in Laemmli Sample buffer (BioRad) and their contents were separated by 10% SDS-PAGE, where the methylation was visualized by immunoblotting with anti-mERG, ERG (sc-271048 Santa Cruz Biotechnology Inc.), and EZH2 (BD Biosciences) antibodies. Parallel control reactions were done in the absence of SAM Master Mixture or in the absence of recombinant PRC2 complex (Active Motif).

### In vitro binding assay by Microscale Thermophoresis (MST)

EZH2 recombinant protein His-tagged (Epigentek, E24031-1) was labeled with Monolith His-Tag Labeling Kit (MO-L008). ERG recombinant protein (Origene, TP308093) at a concentration ranging from 0.4uM to 2.44E-05uM or BSA (0.02uM), was incubated with labeled EZH2 (5 nM fixed concentration) at room temperature for 5 min. Microscale thermophoresis measurements were performed utilizing a Monolith NT.115 instrument (NanoTemper Technologies GmbH, Munchen, Germany). The measurements were performed using Premium capillaries at 30% LED excitation and high MST power.

### RNA analysis

Total RNA was extracted using Trizol (Invitrogen) and Direct-zol RNA-MiniPrep kit (Zymo Research) from both cell line and tissue samples. Quantitative real-time RT-PCR (qRT-PCR) was performed using 20 ng of RNA as template for SYBR Green Fast One-step kit (Qiagen). The level of each gene was calculated by comparing the *C*_*t*_ value in the samples to a standard curve generated from serially diluted RNA from a reference sample and normalizing to the amount of β-actin for cell lines or Rn18s for the tissue samples. Sequences of all PCR primer sets used in the study are shown in Supplementary Table 2, (see Supplementary Info).

### Dual luciferase reporter assay

Cells were plated in 48-well plates and 24 h later transfected with the pGL3-ETS responsive element promoter reporter (Panomics), pGL3-IL-6 promoter reporter (provided by Stephanie Cabarcas), pGL3-NKX3.1 promoter reporter (provided by J. M. Bentel) along with control empty vector pcDNA3.1 or ERG, ∆N-ERG3, ERG-K362A, EZH2, and EZH2-ΔSET expression vectors. Renilla pRLSV40 (Promega) was used as control to monitor transfection efficiency. Luciferase activity was measured after 24 h using the Dual-Luciferase Reporter Assay System (Promega) as previously described. Data are presented as Firefly luciferase activity normalized to the Renilla luciferase activity relative to cells transfected with control vector alone. Reporter assays were performed in triplicate and repeated in three independent experiments.

### Human samples

Tissue samples were taken from patients with organ-confined prostate cancer treated with radical prostatectomy at the Biella Hospital (Biella, Italy) with the approval of the Ethical Committee of the Piedmont region and patient’s written informed consent.

### Chromatin immunoprecipitation (ChIP), ChIP–reChIP, and ChIP_sequencing

Cell line samples (500,000 per IP) or tissue samples (after homogenization) (25 mg per IP) were exposed to formaldehyde (37%) to cross-link protein–DNA complexes and processed as previously described^32,33^. Chromatin extracts were immunoprecipitated with antibodies against ERG (sc-354 X, Santa Cruz Biotechnology Inc.), EZH2 (AC22, Active Motif), acetylated histone H3 (Active Motif), tri-methylated histone H3 at lysine 27 (H3K27me3) (Upstate Biotechnology), SUZ12 (Active Motif), AR (#06-680, Millipore), and IgG (Millipore) as control. For ChIP–reChIP experiments, cells were treated and processed as above. Then, before the second round of immunoprecipitation, beads were eluted with 10 mM DTT at 37 °C for 30 min and immunoprecipitation was carried out as described above. ChIP-sequencing was performed using the anti-mERG antibody in VCaP cells.

Quantitative real-time PCR was performed using KAPA SYBR Fast qPCR kit (KAPABiosystems) and primers spanning the region of interest (Supplementary Table 2). The amount of immunoprecipitated DNA was calculated in reference to a standard curve and normalized to input DNA or subsequently to IgG^33^. For comparison of test and control samples, the amount of DNA was then normalized to the control samples.

### Proliferation, anoikis, migration, and colony formation assays

To assess cell proliferation, cells were plated in 96-well plates at the density of 3 × 10^3^ cells/well for LNCaP and 10 × 10^3^ or 5 × 10^3^ cells/well for VCaP. After 24 h, cells were treated with DMSO as control and different drug concentrations (DZNep, GSK343). For GSK343 treatments, media and drug was replaced every 4 days. The number of viable cells was measured using a colorimetric assay (MTT, Sigma) by adding MTT solution, followed by incibation for 4 h. Then solubilization solution/stop mix was added and the plates were incubated overnight. The optical density (OD) of each well was recorded at 570 nm using a microplate spectrophotometer (BioTek, Beijing). For GSK343 treatments, media and drug was replaced every 4 days. Wells with tissue culture medium but no cells inoculated were used as negative controls. All experiments were repeated in five replicates. The proliferation ratio is analyzed by GraphPad Prism (7.00). Cell survival in anchorage-independent conditions (anoikis assay) was assessed by plating cells in poly-hema-coated 96-well plates. Cell viability was measured using a colorimetric assay (MTT, Sigma) and reading absorbance at 570 nm in microplate reader. Treatment with DZNep was performed by adding the drug or DMSO to the medium during cells plating. Cell migration was assessed using the scratch wound healing assay^34^. Cells were grown to confluence in 12-well plates and maintained overnight in OPTIMEM. After 24 h, scratches were performed on the cell monolayer, complete medium was added to the cultures, and images were taken at time 0 and after 72 h. For clonogenic assay, LNCaP or RWPE (5 × 10^2^ cells/well) or VCaP cells (8 × 10^3^ cells/well) were plated in 12-well plate. When needed after 24 h, cells were treated with DMSO as control or indicated drug concentrations. Colonies were stained with crystal violet and scored. All assays were performed in triplicates and results are represented as mean ± SD from three independent experiments.

### Soft agar assay

VCaP or LNCaP cells were plated (10–30 × 10^3^ cells per dish) in low-grade agar (1.8%) and when indicated DSMO (control), DZNep, or GSK343 was added. After 3–4 weeks, colonies were fixed and stained with 0.01% crystal violet in 20% ethanol and counted. Each experiment was carried out in triplicate and repeated at least three times.

### Sphere-forming assay

For in vitro sphere-forming assay, single-cell suspensions (1 × 10^3^ VCaP cells/well, 2 × 10^3^ LNCaP or RWPE cells/well) were plated in poly-hema coated 6-well plates in serum-free mammary epithelial basal medium (MEBM)^35^. After 10 days, spheres with a diameter greater than 50 µm were counted and sphere colony formation efficiency (SFE) was evaluated according to the following formula: (number of colonies/number of cells inoculated) × 100%). Treatments were performed by adding the indicated drugs to the medium during the sphere-forming assay. Each experiment was carried out in triplicate and repeated at least three times.

### Animals and tumor xenografts

Mice were purchased from the Harlan Laboratories. Mice were maintained under pathogen-free conditions with food and water provided ad libitum and their general health status was monitored daily. For subcutaneous tumor xenografts RWPE1 (5 × 10^6^) stably expressing EV(empty vector), ERG wild-type (ERG), ERG double-mutant (DM), and ERG K362A mutant (K362A) were inoculated with Matrigel (1:1) in the flank of NOD.Cg-Prkdcscid Il2rgtm1Wjl/SzJ (NSG) mice (*n* = 4/group). Tumor growth was monitored every 2 days with a caliper. For subcutaneous tumor xenografts exponentially growing LNCaP cells (3.5 × 10^6^ cells) stably expressing wild-type ERG (ERG), mutant K362A (K362A), or an empty vector (EV) were inoculated in the flank of NSG mice as described above (*n* = 3/group). Tumor growth was monitored every 2 days with a caliper. Tumor xenografts and animal handling were conducted in conformity with the institutional guidelines for animal experimentation and in compliance with national and international policies. Study protocol was approved by the Swiss Cantonal Veterinary Authority (approval number:30010).

### Mouse breeding and in vivo treatment of ERG/PTEN mice

The PbCre4; Pten flox/+ R26LSL;ERG mouse was generously provided by Dr. Charles L. Sawyers^8^. Genotyping was performed using the following primers: R26-TA-WT-3F (5′-TCCCGACAAAACCGAAAATC-3′), R26-WT-3R (5′-AAGCACGTTTCCGACTTGAG-3′), ERG Ex7F (5′-CAAAACTCTCCACGGTTAATGC-3′) and ERG Ex10R (5′-GCACTGTGGAAGGAGATGGT-3′), and with WT band of 468 bp and a targeted band of 205 bp. Pb-Cre4; Rosa26ERG/+ and Pb-Cre4. Rosa26ERG/ERG mice were generated through standard mouse breeding. After generation of Rosa26ERG/ERG homozygous mice, subsequent crosses involved Pb-Cre4; Rosa26ERG/ERG males with Rosa26ERG/ERG females that generated a 1:1 ratio of Cre+ and Cre− mice. Pb-Cre4; Rosa26ERG/+ and Pb-Cre4; Rosa26ERG/ERG mice were generated through standard mouse breeding and after generation of Rosa26ERG/ERG homozygous mice, subsequent crosses involved Pb-Cre4. To generate double-homozygous mice for ERG and Pten we crossed Pb-Cre4; Rosa26ERG/ERG mice to Ptenflox/flox mice to obtain Pb-Cre4;.Rosa26 ERG ^flox/flox^;Pten ^flox/flox^ (ERG/PTEN mice).

To study the effect of the EZH2 inhibitor GSK343 in vivo, ERG/PTEN mice 25 weeks old (*n* = 5) were injected intraperitoneally (15 mg/kg) three times weekly for 2 weeks or with vehicle (*n* = 5) for control. After 2 weeks, the mice were sacrificed and the prostates were surgically excised, weighed, photographed, and sectioned. Sections of excised prostates embedded in paraffin were subjected to histological and immunohistochemical analysis. IHC were independently evaluated by two investigators to assign scores to multiple sections. All the protocols (breeding and treatment) were approved by the Swiss Cantonal Veterinary Authority (approval number:31524) and were conducted in conformity with the institutional guidelines for animal experimentation and in compliance with national and international policies.

### Nuclear/cytoplasm fractionation

For nuclear/cytoplasm fractionation, prostates from ERG/PTEN or wild-type mice were dissected, minced into small pieces, and incubated in Hanks Balanced Salt solution (Sigma: H9394) supplemented with collagenase (1 mg/ml, Sigma: C0130) and Dispase II (1 mg/ml, Sigma: D4693) for 2–3 h. Lysates from nuclear and cytoplasmic fraction were collected using the NE-PER nuclear and cytoplasmatic extraction reagents, (ThermoFisher cat 78833).

### Chick chorioallantoic membrane (CAM) assay

The CAM assay system was used to assess the ability of VCaP cells to migrate, disseminate to blood vessels, and metastasize to the liver in vivo as previously described^36,37^. Fresh fertilized eggs (Animalco AG; Staufen, Switzerland) were incubated at 37.5 °C (60–62% humidity) for 3 days. On day 3 CAM dropping was done by making a small window in the shell under aseptic conditions. The window was resealed with adhesive tape and eggs returned to the incubator until day 9 of chick embryo development. On day 9, 2.5 × 10^6^ cells (*n* = 8) were implanted onto the CAM. The eggs were resealed and returned to the incubator until day 14. On day 14 of the experiment, lower CAM and/or liver were dissected to evaluate cell migration and liver metastasis. DNA extraction was done using Qiagen DNeasy Blood & Tissue Kit (Catalog # 69504). The extent of metastatic human cells to the liver was evaluated by assessing enrichment of human DNA in the chick embryo by real-time PCR^38^. To detect human cells in the chick tissues, primers specific for the human Dloop sequences (sense: 5′-CTAAATAGCCCACACGTTCC-3′; and antisense: 5′-TAGGATGAGGCAGGAATCAA-3′) were used to amplify human DNA present in genomic DNA extracted from chicken liver tissue. PCR reaction contained DNA, 2x Mastermix (SYBR FAST qPCR Master Mix, Kapabiosystems), and forward and reverse primers (400 nM) in a 10 μl final volume. PCR was carried out at 95 °C for 3 min (1 cycle), 95 °C for 3 s, and 60 °C for 30 s (40 cycles). A quantitative measure of amplifiable chick DNA was also obtained through amplification of the chick DNA with primers (sense: 5′-TACTTCATGACCAGTCTCAGG-3′; antisense: 5′- AGTTCAGGAGTTATGCATGG-3′) specific to Chicken Dloop region using the same PCR conditions as described above. Data analysis was based on comparison of the amplification signals of the species specific highly conserved Dloop regions. The human Dloop signal was normalized against the relative quantity of chicken Dloop signal and expressed as Δ*Ct* = (*Ct*_*Human*_ − *Ct*_*Chicken*_). The changes in *Human Dloop* signal relative to total genomic DNA were expressed as ΔΔ*Ct* = Δ*Ct*_control_ − Δ*Ct*_treatment_. Relative changes were then calculated as 2^-ΔΔ*CT*^. For drug treatments (a) DZNep: cells were treated in vitro with DMSO or drug (10 μM) for 48 h. After treatment cells were collected, counted, and implanted (2.5 × 10^6^ cells in 15 μl) onto the CAM as described above. (b) For GSK343: after cell explant, CAM was treated with DMSO or GSK 343 (5 mg/kg) on day 0 and day 2. For in vivo imaging of cell dissemination in CAM, confocal microscopy was used. VCaP cells were stained with green fluorescent cell linker dye (PKH67, Sigma Aldrich) and CAM assay was performed as described above. On day 14 of embryonic development, immediately prior to harvesting, 50 μg of Rhodamine-conjugated *Lens Culinaris* Agglutinin (LCA) (Vector Laboratories Inc., Burlingame CA) was injected i.v. to label the chicken vasculature as previously described^37,39^. Lower CAM was dissected and fixed in 10% NFB (Neutral Formalin Buffer, Thermo Scientific). The tissue was mounted on slide with mounting medium (Thermo Scientific Shandon™ Immu-Mount™) and representative images were taken. The imaging was performed using a Leica TCS SP5 confocal microscope, using a HCX PL Apo 40X/1.25 N.A. oil immersion objective. Z-stacks with a total thickness of 10–15 μm were acquired every 2 μm. To reconfirm the quantification data from metastases assay. 8 images per group with above settings were manually quantified for cell number.

### Immunohistochemical staining (IHC) on FFPE (Formalin Fixed, Paraffin Embedded)

Sections from mice were examined by IHC to assess ERG, EZH2 and pEZH2S21, Ki-67 expression on 4 mm FFPE prostatectomy sections. Immunohistochemistry was carried out using anti-ERG rabbit monoclonal (1:50 dilution, Abcam (ab92513)), anti-EZH2 (D2C9) Rb Monoclonal (1:400 dilution, CST #5246), anti-pEZH2S21 (1:100 dilution, IHC-00388 Bethyl Laboratories), and anti Ki67 (SP6) (RTU Lab Vision #RT-9106-R7 (7 ml)) antibodies. Staining was independently scored by two investigators. Immunohistochemistry was done by dewaxing and rehydrating the sections followed by antigen retrieval with water bath 98 C° 20’pH6 citrate buffer or 9 EDTA buffer. Peroxidase blocking of the sections was done for 10 min at room temperature (3% H_2_O_2_). After three times washing with PBS–Tween, sections were incubated in protein block serum free (DAKO, X0909) for 10 min. They were then incubated in antibody solution for 1 h at room temperature at the right dilution in antibodies diluent. Sections were washed in PBS–Tween. As secondary antibody RTU Biotinilated anti rabbit (Vector P-9100) for 30 min at room temperature was used. Then Vectastain ABC Kit (Vector PK-6100) (A 1:150 + B 1:150 in PBS) for 30 min at room temperature was used. Then Vectastain ABC Kit (Vector PK-6100) (A 1:150 + B 1:150 in PBS) was used for 30 min at room temperature. Nuclear visualization was carried out with DAB: ImmPACT DAB Peroxidase (HRP) Substrate (Vector Lab SK-4105) for 4 min at room temperature. Counterstaining was done with hematoxylin for 1 min at room temperature. Sections were dehydrated and stabilized with mounting medium and images were taken by optical microscope.

### Ex vivo sphere-forming assay

For ex vivo sphere-forming assay (SFA), Pb-Cre4;.Rosa26 ERG ^flox/flox^;Pten ^flox/flox^ (ERG/PTEN) transgenic mouse prostates from 27- and 33-week-old mice were dissected and minced into small pieces in Hanks Balanced Salt solution (Sigma: H9394) supplemented with 1 mg/ml of collagenase (Sigma: C0130) and 1 mg/ml of Dispase II (Sigma: D4693) for 3 h. Cell suspension was passed through a 40 µm cell strainer (Falcon: 352340) to collect single cells. Further cells were centrifuged (2000 rpm for 5 min), washed (PBS, 2 times), and plated in poly-hema coated 6-well plates in serum-free mammary epithelial basal medium (MEBM)1 at a concentration of 5000 cells/well. Medium was replenished every 3 days. After 10 days, spheres with a diameter greater than 50 µm were counted. Treatments were performed by adding the indicated drugs to medium 24h after plating the cells^2^. Each condition was carried out in triplicate.

### Gene expression profiling

RNA was collected from LNCaP with stable expression of wild-type ERG (ERG-WT), ERG mutant (ERG-K362A), or empty vector (EV). We performed 8x60k Sure Print G3 Human GE Arrays. RNA was amplified, labeled, and hybridized according to the two-color microarray-based gene expression analysis protocol (Agilent Technologies). Slides were scanned with the dual-laser scanner Agilent G2505B and analyzed as described. Differentially expressed genes were obtained by selecting probes with absolute log2 fold change >0.37 and adjusted *P* value <0.05. Data are MIAME-compliant and have been deposited in GEO (GSE71329) https://www.ncbi.nlm.nih.gov/geo/query/acc.cgi?acc.

### Genome wide analysis of ERG and EZH2 occupancy

To determine the genome-wide co-localization of EZH2 and ERG complexes, we analyzed ChIP-seq datasets (GSE28951) performed in VCaP cells^20^. Reads for EZH2, ERG, and Input were mapped to the hg19 human genome assembly using bowtie and only uniquely mapped reads were kept^40^. Using MACS software^41^, we elicited 14,780 peaks from the EZH2 data set and 48,274 peaks from the ERG data set. Peaks were annotated with our software (peak-tool) using all regions of the gencode-v19 database^42^. The tool reported the presence of peaks in gene-promoters, gene-bodies, and enhancers and their distances to the TSSs. For co-localization analysis of ERG EZH2, overlapping peaks within ≤1-kb window were scored and visualized using Venn diagram generator tools (http://www.pangloss.com/seidel/Protocols/venn.cgi). Ab-initio motif enrichment analysis in the ERG proximal EZH2 peaks was done using MEME tool^43^. Peak intensity profiles and heatmaps (region wise as well as total) were generated using deepTools suite^44^. The gene-promoter occupied by ERG identified by peak analysis was crossed with lists of genes modulated in LNCaP cells by ERG and ERGK362A using venn diagram generator tools as described above.

Publicly available datasets were downloaded from GEO, processed, and analyzed. The Sboner dataset, Weill Cornell Medical College (GSE16560) includes 281 primary prostate tumors with indication of the ERG fusion status, vital status, and overall follow-up^24^. The TCGA (The Cancer Genome Atlas) dataset (downloaded from http://gdac.broadinstitute.org/) includes 497 primary prostate tumors. The Prostate Adenocarcinoma dataset, (cBioPortal: Michigan, Nature 2012)^45^ (GSE35988) contains 59 primary and 35 Castration resistant, metastatic prostate cancer. All the statistical and bioinformatic analyses were performed in R environment. The differential expression analysis in microarray and RNA-seq databases were retrieved according to Limma and DESeq pipeline, respectively. To extract the top ranking ERG/EZH2 signature of 50 genes (DE_topERG/EZH2), genes differentially expressed between ERG-positive and ERG-negative samples in Sboner dataset were assessed with a *p*-value cut-off of 0.1. Then, this list was merged with the ERG/EZH2 list and *n* = 50 genes were extracted and investigated in Mitchigan and TCGA datasets. For functional annotation, the enrichment analysis of the co-occupied genes (ERG–EZH2) was based on the DisGeNET platform *enrichDGN (disease gene net, tool from the DOSE R package)* and was performed through the DOSE R package. Additional tools included Enrichr (http://amp.pharm.mssm.edu/Enrichr/). Comparison among different classes of samples, clustering analysis was performed using BRB-ArrayTools (developed by Dr. Richard Simon and BRB-ArrayTools Development Team) and R statistical computing environment. Clustering was performed using the one minus correlation metric and average linkage after genes were centered and scaled. Principal Component Analysis was performed using the PCAtools. Gene set enrichment analysis (GSEA) was performed using the tool from the Broad Institute (http://www.broadinstitute.org/gsea/). *T*-test was used as metric for ranking genes after 1000 permutations.

### Computational modeling

#### Generation of ERGi and peptide models

We focused our molecular dynamic simulations (MDS) on the ERG region from residue 272 to residue 412. Based on the study by Regan et al.^18^ this region, named ERGi, represents the construct with the maximal auto-inhibitory effect. The atomistic structure of the auto-inhibited ETS DNA-binding domain (DBD) of ERG in complex with DNA solved by X-ray and deposited into the protein data bank (PDB) with the code 4IRI does not exactly correspond to the 272–388 construct; the deposited structure starts with Gly292 and ends with His385. Also, the backbone chemical shifts deposited in the BRBM database (accession number 19137, http://www.bmrb.wisc.edu) were assigned for fragment from Gln272 to Glu388. Therefore, we built our ERG molecular model using the ERG X-ray structures identified by the pdb codes 4IRI, 4IRH, and 2NNY and the backbone chemical shifts deposited in the BRMB database as experimental constraints. These data were used to build an initial model with the CS23D2.0 web-server, which was subsequently refined using CamShift^46^ and ALMOST^47^ to produce the final models of ERGi with either methylated or non-methylated K362.

The structure of the peptide, corresponding to residues from P351 to L376, used to generate the anti-mERG antibody was extracted from the ERG structure identified by the PDB code 4IRI^18^ and modeled in its methylated and non-methylated form.

### Molecular dynamics (MD) simulations

The generated ERGi models were simulated in the NPT ensemble (298.5 K and 1 Atm) for 5 μs using the pmemd.cuda MD engine available in Amber16^48^. The peptide models were simulated for 120 ns. To improve sampling of the conformational space, the simulation was replicated 9 times for a total simulation time exceeding 1 μs as described by Perez et al.^49^.

All models were prepared according to an identical protocol. The protein was immersed in a water box with a minimal thickness of 10 Å from the protein surface and a suitable number of ions (two Cl^-^ ions for the ERGi models and three for the peptides) were added to the system to ensure electrostatic neutrality.

The ff14SB^50^ version of the Amber force field was used to describe the protein; the TIP3P water model^51^ for the water and the parameters proposed by Cheatam^52^ and coworkers for the chlorine ions were applied, respectively. Amber parameters for methylated lysine were taken from Papamokos et al.^53^. The system was minimized and then slowly heated (over 150 ps) to a temperature of 300 K, while the pressure was fixed to a value of 1 atm. Temperature and pressure were regulated using the Langevin thermostat and the Monte Carlo barostat as implemented in the pmemd.cuda code available in the Amber16 distribution. Once the system was equilibrated, production run was done with a time step of 0.002 fs (bonds involving hydrogen atoms were constrained using the SHAKE algorithm). Van der Waals and short-range electrostatic interactions were estimated with a cut-off of 8 Å, while the long-range electrostatic interactions were taken into account using the particle mesh Ewald method.

### Trajectory analysis

The solvent accessible surface area (SASA) was calculated using the molsurf program available inside AmberTools16.Cluster and RMSF analysis was performed with the g_cluster and the g_rmsf tools from gromacs 4.6. Cluster analysis was performed applying the default set-up and RMSD cut-off of 2 Å for the protein and 1 Å for the peptide. Contact analysis was performed using the g contacts program developed by Bau and Grubmuller^54^. The inter-molecular distance between selected residues (Leu320 and Ala413) was calculated in all systems over the simulation time to estimate the degree of divergence relative to the active DNA-bound ERG (4iri) structure defined by X-ray crystallography. For evolution analysis of the secondary structure, the local secondary structure was determined over the simulation time using STRIDE software as implemented in the Timeline tool available in the VMD software (version 1.9.4).

### Statistical analysis

GraphPad Prism version 7.00 (GraphPad Software) and microsoft excel were used for analysis. An unpaired, two-tailed independent Student’s *t* test with unequal variance assumption was performed to analyze the statistical significance of differential findings between experimental groups. For ERG/EZH2 co-regulated gene signature *T*-test was used as metric for ranking genes after 1000 permutations. Error bars represent mean (SD) if not stated differently. For the Scatter plot of the LogRatios of genes, the regression line was calculated by means of the lm function (regression line coefficient = 0.44). Numbers of replicates (*n*-values) are specified for each experiment.

### Reporting summary

Further information on research design is available in the Nature Research Reporting Summary linked to this article.

## Supplementary information

Supplementary Information

Description of Additional Supplementary Files

Supplementary Data 1

Supplementary Data 2

Supplementary Data 3

Reporting Summary

## Data Availability

In house Gene expression profiling data sets were deposited in the GEO database (GSE71329) and are accessible at: https://www.ncbi.nlm.nih.gov/geo/query/acc.cgi?acc=GSE71329. In house Chip sequencing data (mERG) have been deposited in the GEO database (GSE159471) and are accessible at https://www.ncbi.nlm.nih.gov/geo/query/acc.cgi?acc=GSE159471. Publicly available ChIP1018 seq datasets were downloaded from GEO database (GSE28951) and can be accessed at https://www.ncbi.nlm.nih.gov/geo/query/acc.cgi?acc=GSE28951. Publicly available databases of transcriptomic data retrieved are: Sboner dataset, Weill Cornell Medical College (GSE16560) accessible at https://www.ncbi.nlm.nih.gov/geo/query/acc.cgi?acc=GSE16560; TCGA dataset accessible at https://portal.gdc.cancer.gov/; Prostate Adenocarcinoma dataset, accessible at https://www.ncbi.nlm.nih.gov/geo/query/acc.cgi?acc=GSE35988. The remaining data are available within the Article or Supplementary Information. Source data are provided with this paper.

## Source data

Source Data
