# Supplementary material for: EZH2-induced lysine K362 methylation enhances TMPRSS2-ERG oncogenic activity in prostate cancer
Source: Nat Commun. 2021 Jul 6;12:4147. doi: 10.1038/s41467-021-24380-6 (PMC8260656; doi:10.1038/s41467-021-24380-6)
Supplement: Supplementary file 1 — Supplementary Information [file 41467_2021_24380_MOESM1_ESM.pdf]

Supplementary information:

Supplementary Figures

Zoma ,Curti et al. 2021

Corresponding author: Giuseppina M Carbone pina.carbone@ior.usi.ch

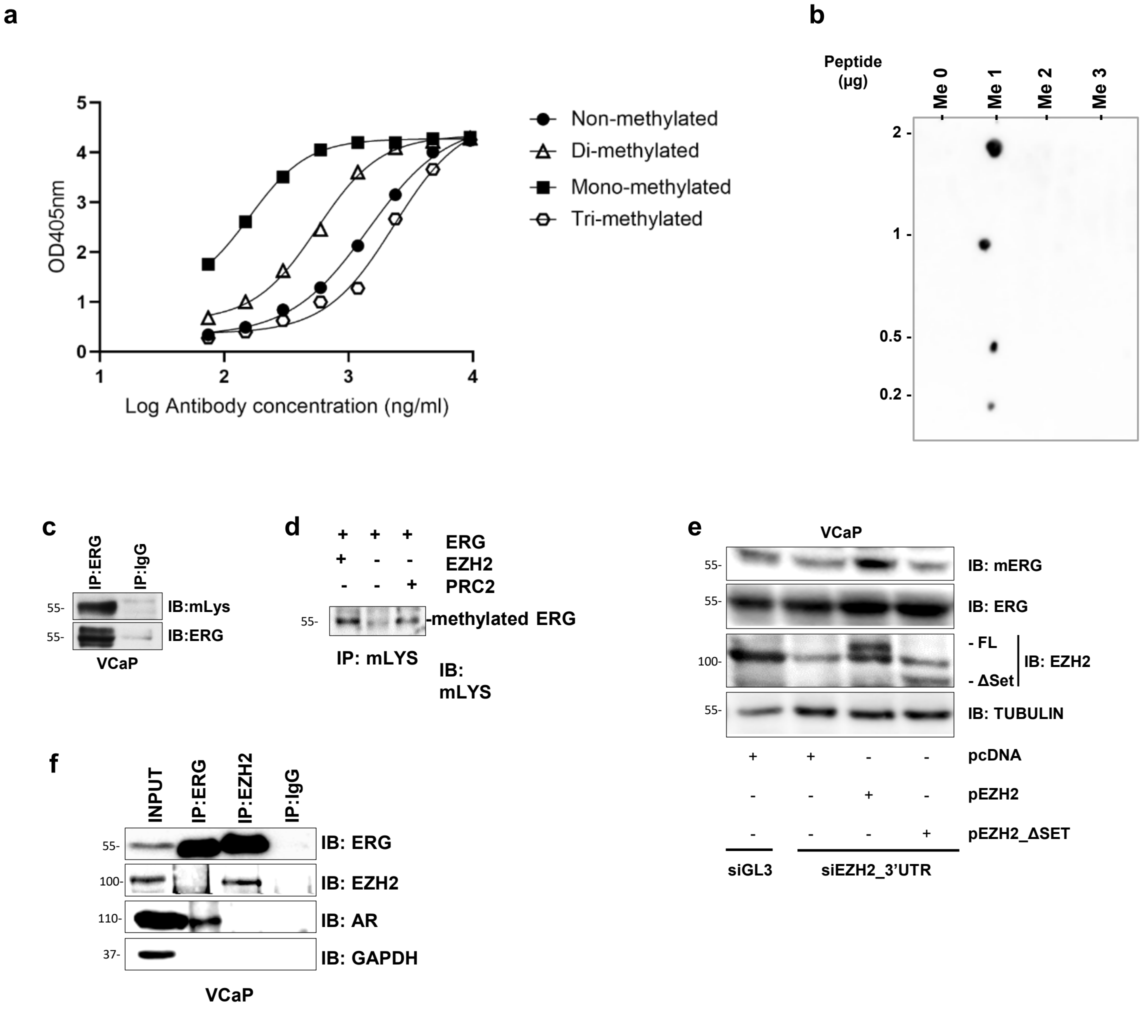

Supplementary Figure 1. Detection of K362 methylated ERG and ERG/EZH2 interaction in human cells. a, Binding of mERG antibody to ERG peptides (non-, mono- di- and tri- methylated), determined by enzyme-linked immunosorbent assay (ELISA). Plates were coated with biotinylated peptides and incubated with serially diluted mERG Ab. Absorbance was read at 405 nm. **b**, Detection of non-methylated and mono-, di- and tri- methylated ERG peptide with the anti-mERG antibody by immunoblotting. **c**, Immunoprecipitation (IP) with anti-ERG antibody followed by IB with anti-methyl-lysine (mLys) antibody (n=2). **d,** In vitro methylation assay with recombinant ERG incubated with EZH2 or PCR2 complex followed by IP with anti-methyllysine (mLys) antibody (n=2). **e**, mERG in EZH2 depleted (siEZH2-3'UTR) VCaP cells and reconstituted with WT or mutant EZH2 lacking the catalytic domain (pEZH2_ΔSET) (n=2). **f,** ERG, AR and EZH2 interaction assessed by IP in VCaP cells (n=2). Molecular weights are indicated in kilodaltons (kDa).Source data are provided as a Source Data File.

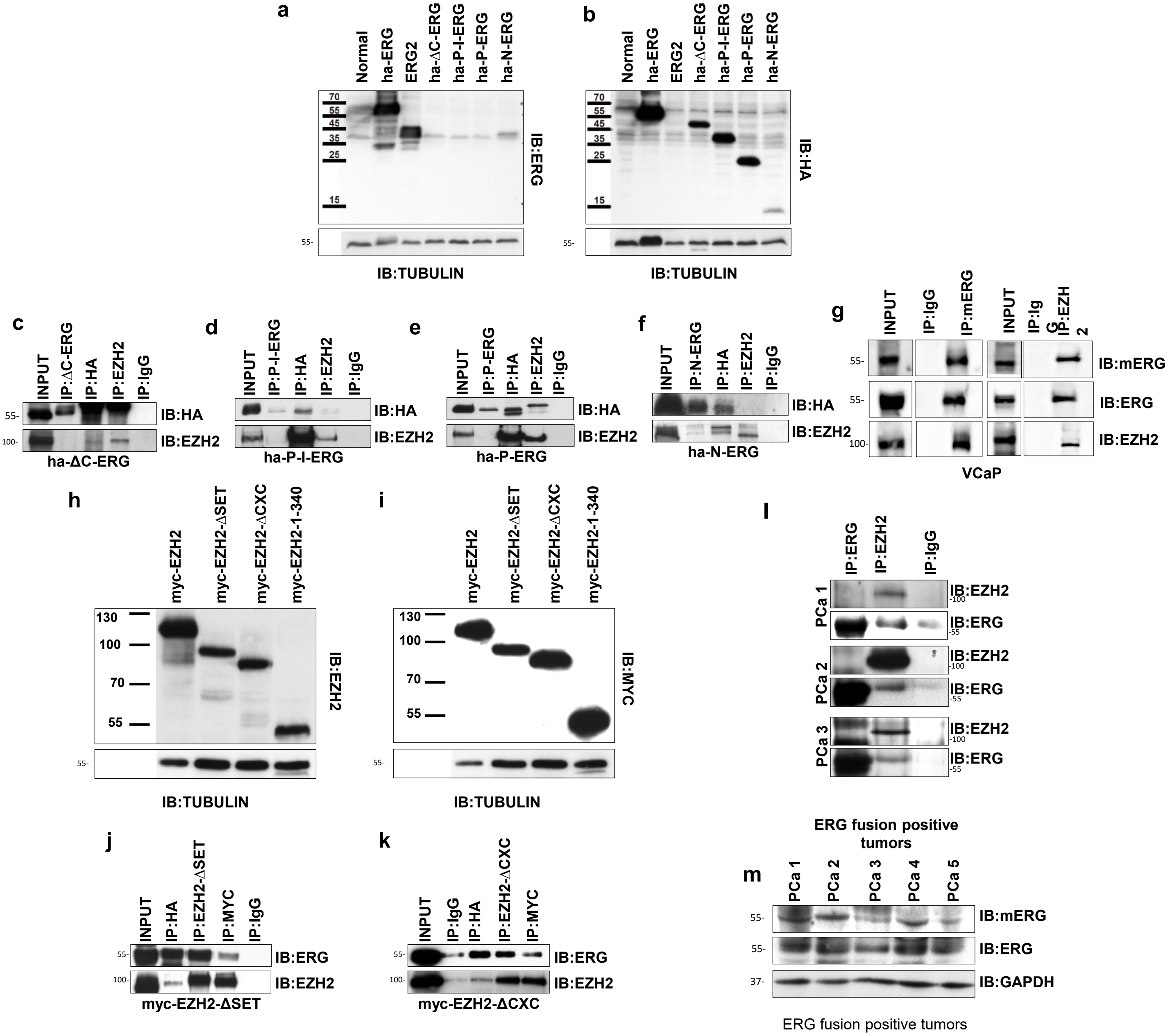

Supplementary Figure 2. Identification of ERG and EZH2 interacting domains. a-b, Immunodetection of the full length and truncated ERG constructs using anti-ERG (**a**) and anti-HA (**b**) antibodies (n=2). **c-f**, Binding of Ha-ΔC-ERG (**c**), Ha-P-I-ERG (**d**), Ha-P-ERG (**e**), Ha-N-ERG (**f**) to EZH2 assessed by co-immunoprecipitation in PC3 cells transiently transfected with the truncated ERG constructs (n=2). **g**, Immunoprecipitation of mERG and EZH2 in VCAP cells and immunoblots with the indicated Ab (n=2). **h-i**, Immunodetection of the full length and truncated EZH2 constructs using anti-EZH2 (**h**) and anti-Myc (**i**) antibodies (n=2). **j-k**, Binding of Myc-EZH2-ΔSET (**j**), Myc-EZH2-ΔCXC (**k**) to Ha-ERG assessed by co-immunoprecipitation in PC3 cells transiently transfected with the truncated EZH2 constructs along with ERG plasmid (n=2). **l**, ERG, mERG and EZH2 interaction in human ERG fusion positive prostate tumors determined by IP followed by IB with the indicated antibodies (n=2). Methyl ERG was detected with pan-methyl Lysine (mLys) Ab (n=2). **m**, Detection of mERG by immunoblotting in ERG fusion positive human prostate tumors (n=2). Molecular weights are indicated in kilodaltons (kDa).Source data are provided as a Source Data File.

a

Contact Frequency		
AA	me-K362	K362
S315	≤ 0.1	0.43
G316	0.63	0.43
Q317	0.49	≤ 0.1
I318	0.65	0.41
W358	0.93	0.99
G359	0.41	0.92
E360	0.19	0.48
R361	1.00	1.00
S363	1.00	1.00
K364	≤ 0.1	0.94
M367	0.28	0.68
H409	0.11	≤ 0.1
P410	0.16	≤ 0.1
P411	0.19	≤ 0.1
**E412**	**0.22**	**0.72**

b

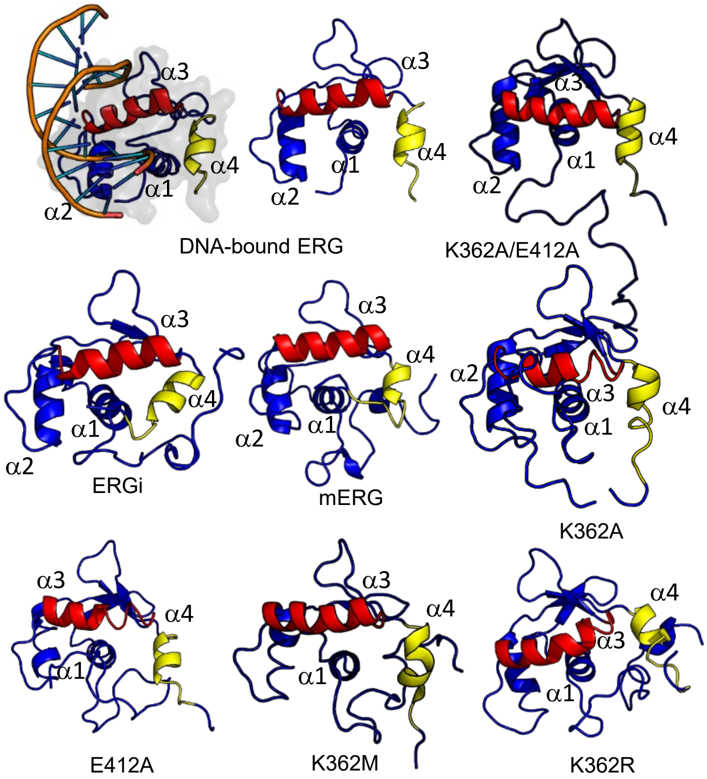

Supplementary Figure 3. K362 methylation alters intramolecular contacts. a, Intramolecular contact frequency of methylated and non-methylated K362 in the ERGi domain. Only contact frequencies > 0.1 are reported. **b**, Extracted structures of uninhibited (DNA-bound ERG), auto-inhibited (ERGi), methylated (mERG) and mutated ERG constructs (K362A, E412, K362M, K362R) by molecular dynamic simulation.

Supplementary Figure 4, Zoma, Curti et al.

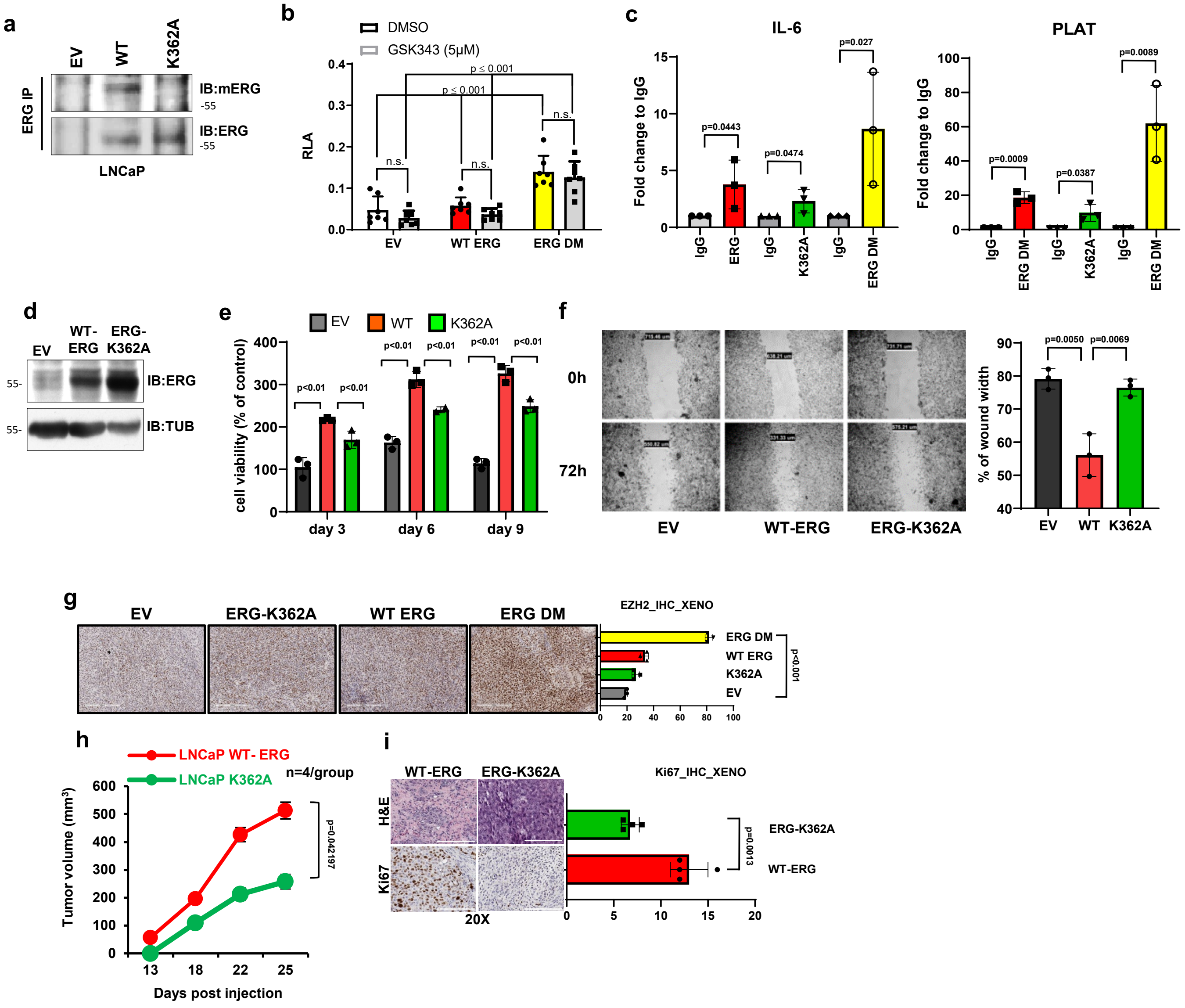

Supplementary Figure 4. K362 methylation enhances ERG oncogenic activity. a, Detection of ERG and mERG in LNCaP cells expressing WT or K362A ERG by IP followed by IB with indicated antibodies (n=2). **b**, Luciferase activity of the ETS responsive reporter in RWPE1 cells expressing WT, K362A, K362A/E412A ERG or empty vector (EV) incubated with DMSO or GSK343(5μM). not significant (n.s.). **c**, Binding of HA-tagged WT and mutants (K362A and ERG DM) ERG to IL-6 (left) and PLAT (right) promoter in RWPE1 cells by ChIP-qPCR with anti-HA antibody. **d**, Stable expression levels of WT and K362A ERG in LNCaP cells. EV, LNCaP expressing control empty vector (n=2). **e**, Survival in anoikis of LNCaP cells stably expressing WT, K362A ERG or an empty vector (EV). Cell viability at the indicated times is presented as percentage relative to day 0. **f**, Cell migration by wound healing assay in LNCaP cells stably expressing WT or ERG-K362A. Differences in wound width are shown as percentage relative to T0 (right). **g**, Histological and IHC scores of EZH2 in tumor xenografts from EV,WT-ERG or ERG-K362A and ERG DM RWPE1 stable cells. *Right*, IHC scores of EZH2 in indicated RWPE1 xenografts (n=4/group). **h**, Growth of xenografts of LNCaP cells stably expressing wild type ERG (WT-ERG) or mutant K362A (ERG-K362A) injected in NSG mice (n=4/group). **i**, Histological and IHC scores of Ki67 in tumor xenografts from WT-ERG or ERG-K362A LNCaP stable cells. Scale bars represent 200μm. *Right*, IHC scores of Ki67 in indicated LNCaP xenografts (n=4/group). All error bars, mean ± s.d. (n=3, technical replicates). P-values were determined by one-way ANOVA test. Molecular weights are indicated in kilodaltons (kDa).Source data are provided as a Source Data File.

Supplementary Figure 5,
Zoma, Curti et al.

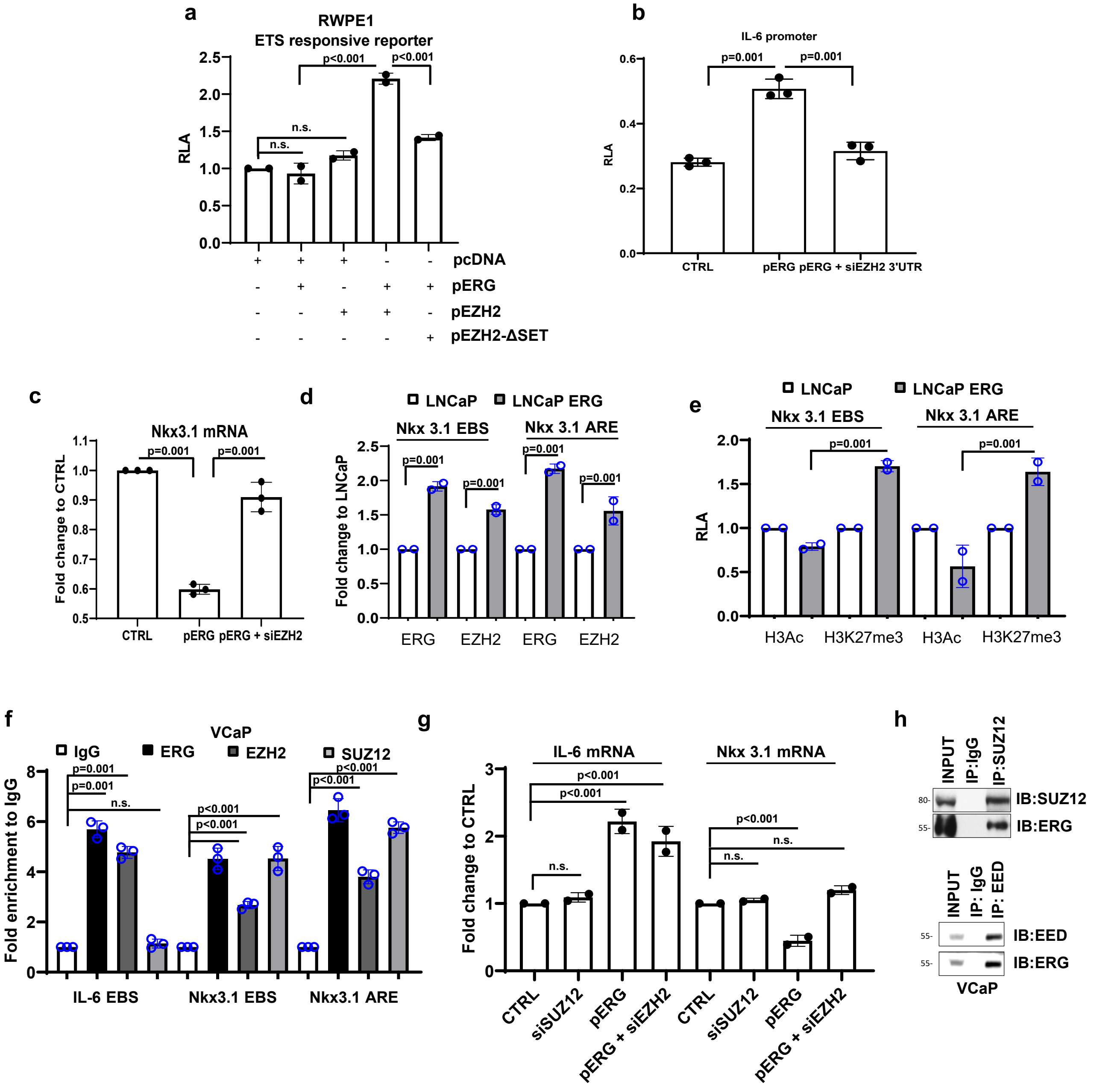

Supplementary Figure 5. EZH2 enhances ERG transcriptional activity a, ETS responsive reporter activity in RWPE1 **(left) and LNCaP (right),** transfected with the indicated vectors including WT-EZH2 and EZH2-ΔSET. **b**, IL-6 luciferase reporter assay after ERG over-expression and EZH2 knockdown. **c**, Nkx3.1 mRNA evaluated by qRT-PCR in LNCaP cells 48 h after ERG over-expression and EZH2 knockdown. **d**, ERG and EZH2 occupancy at Nkx3.1 locus in LNCaP and LNCaP-ERG cells evaluated by ChIP in the regions encompassing the ETS binding site (EBS) and androgen responsive element (ARE). **e**, H3Ac and H3K27me3 at Nkx3.1 promoter in LNCaP and LNCaP-ERG cells. **f,** ERG, EZH2 and SUZ12 occupancy at the IL-6 and Nkx3.1 promoters in VCaP cells. **g**, qRT-PCR analysis of IL-6 and Nkx3.1 mRNA in LNCaP cells 48 h after transfection with the indicated plasmids and siRNAs. n.s., not significant. **h**, Immunoprecipitation of SUZ12 (upper) and EED (lower) in VCaP cells and immunoblots with ERG Ab (n=2). All error bars, mean ± s.d. (n=3, technical replicates). P-values were determined by one-way ANOVA test. Molecular weights are indicated in kilodaltons (kDa). Source data are provided as a Source Data File.

Supplementary Figure 6, Zoma, Curti et al.

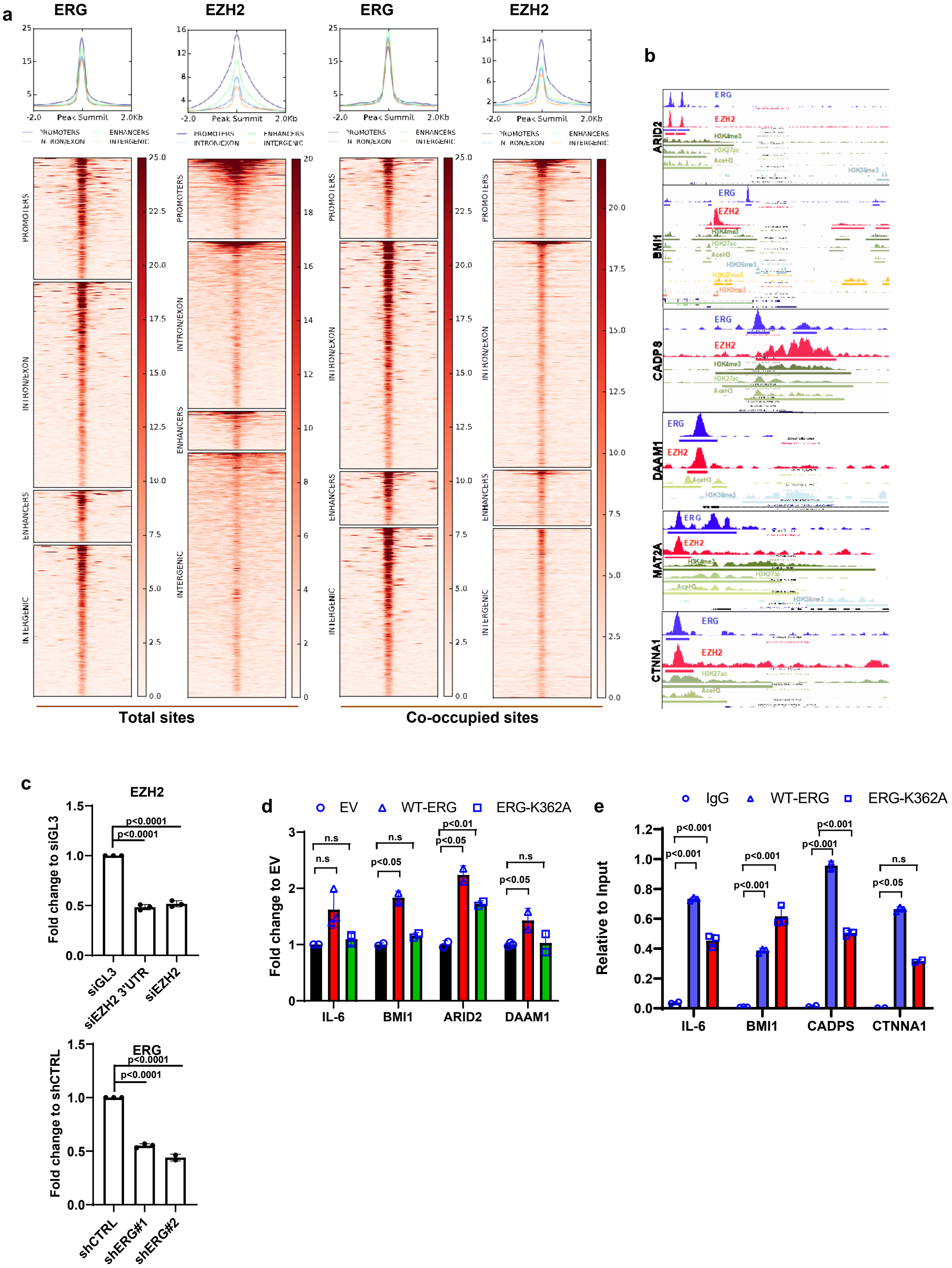

Supplementary Figure 6. Genomic occupancy of ERG and EZH2 and distribution analysis of activating and repressive histone marks in co-occupied regions. a, ChIP-Seq occupancy profiles and density plots of ERG and EZH2 binding events at total sites (left) and ERG/EZH2 co-occupied sites (right) for the indicated genomic regions. **b**, Representative ChIP-Seq occupancy profiles of ERG, EZH2 and indicated histone marks at the promoters of selected ERG/EZH2 co-occupied targets in VCaP cells. Genomic binding at these sites was validated by ChIP-reChIP shown in Figure 5H. **c**, Level of EZH2 and ERG in VCaP cells after knockdown with the indicated siRNAs or shRNAs. **d**, Expression of ERG/EZH2 co-occupied genes evaluated by qRT-PCR in LNCaP cells expressing EV, WT-ERG and ERG-K362A. **e**, ChIP analysis of ERG occupancy at the indicated ERG/EZH2 targeted promoters in LNCaP cells stably expressing WT-ERG or ERG-K362A. Data are presented as fold enrichment relative to input. All error bars, mean ± s.d. (n=3, technical replicates). P-values were determined by one-way ANOVA test. Source data are provided as a Source Data File.

Supplementary Figure 7,
Zoma, Curti et al.

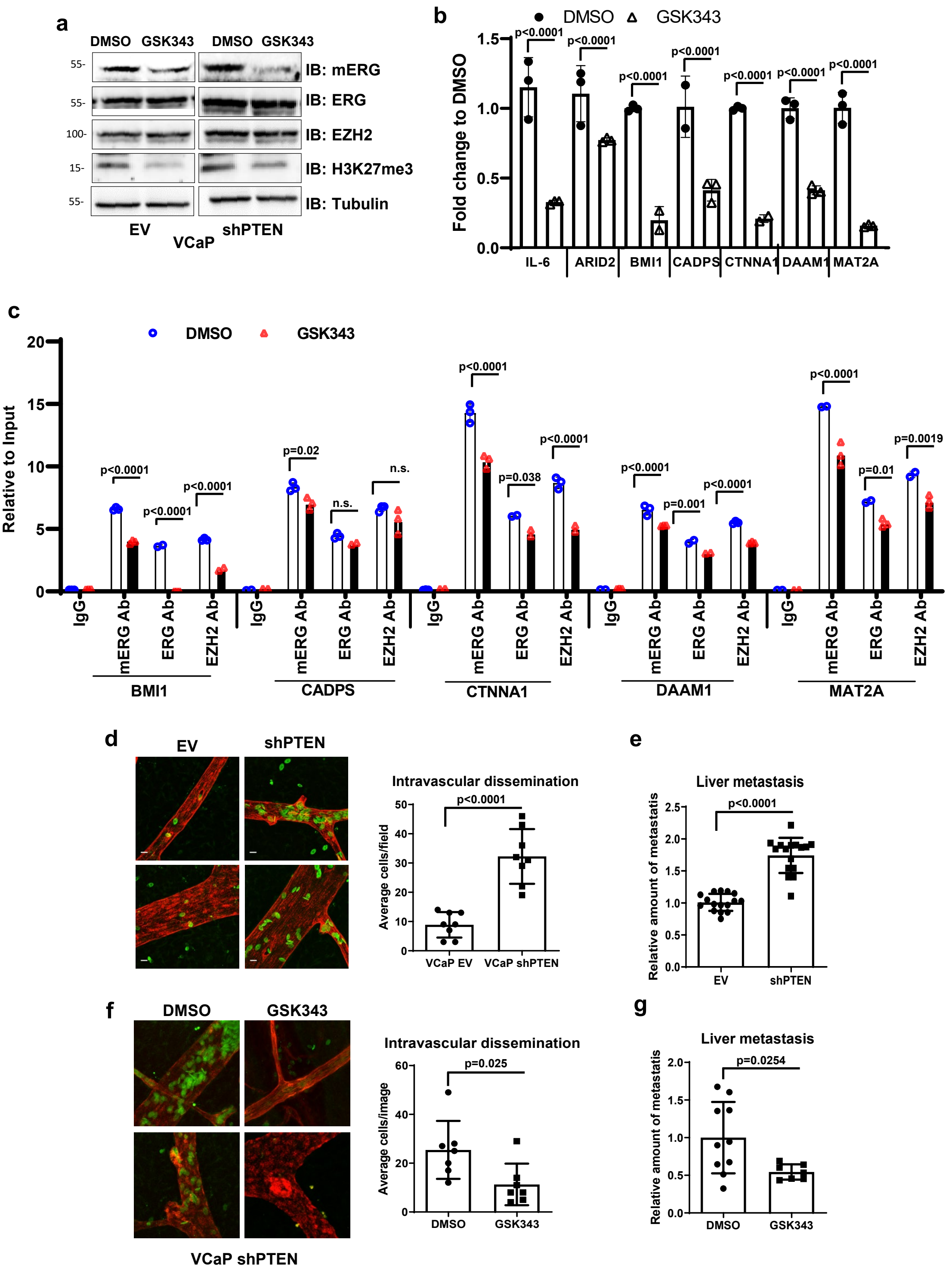

Supplementary Figure 7. PTEN knockdown enhances ERG transcriptional and oncogenic activity and the EZH2 inhibitors GSK343 reverts these effect. a, Immunoblots of mERG and the indicated proteins in control (EV) and stable PTEN knockdown (shPTEN) VCaP cells treated with GSK343 (5µM) or DMSO (n=2). **b**, Expression of indicated ERG/EZH2 targets in control (DMSO) and GSK343 treated VCAPshPTEN cells. **c**, ERG, mERG and EZH2 occupancy at the indicated promoters in VCaPshPTEN cells after treatment for 24 h with DMSO or GSK343 (5µM). **d**, Intravascular dissemination of VCaP-shPTEN and control (EV) VCaP cells in CAM assay. *Right*, quantification of average number of intravascular cells/field. *Left,* Representative images. Red, chicken endothelial cells; green, cancer cells. **e**, PCR-based quantification of metastatic VCaP-shPTEN and control (EV) VCaP cells in chicken embryo liver (n=8) in CAM assays. **f**, Intravascular dissemination of VCaP-shPTEN cells treated with DMSO or GSK343 (10µM) in CAM assay. Left, Representative images of intravascular dissemination formation by VCaP-shPTEN. *Right*, quantification of average number of intravascular cells/field. Red, chicken endothelial cells; green, cancer cells. **g**, PCR-based quantification of metastatic VCaP-shPTEN cells treated with DMSO or GSK343 (10µM) in chicken embryo liver (n=8) in CAM assays. Scale bar = 20µm. All error bars, mean ± s.d. (n=3, technical replicates). P-values were determined by one-way ANOVA test. Molecular weights are indicated in kilodaltons (kDa).Source data are provided as a Source Data File.

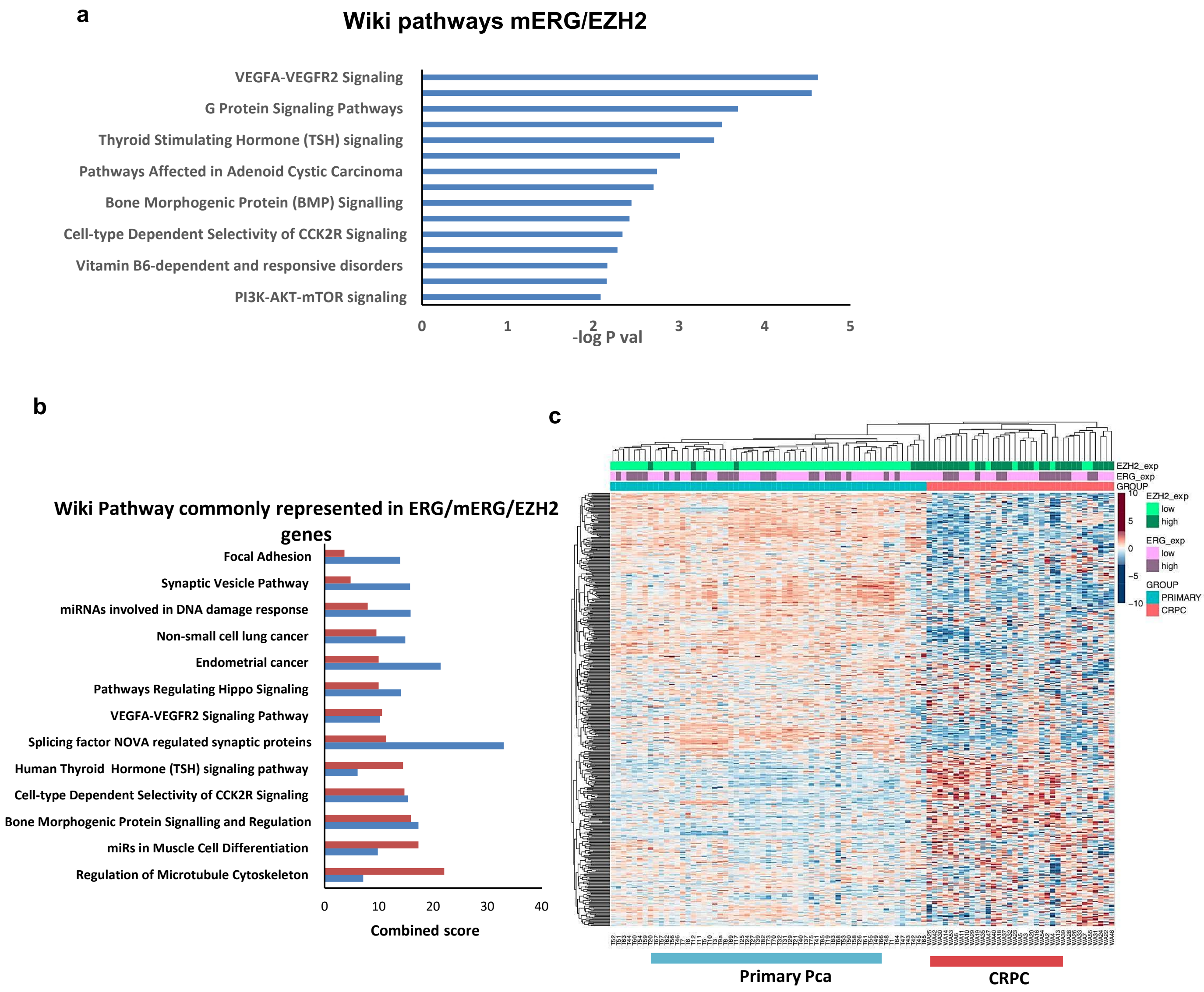

Supplementary Figure 8. mERG/EZH2 co-regulated genes are expressed in aggressive prostate tumors. a, Top pathways enriched in the list of genes co-occupied by mERG/EZH2 (GS_mERG/EZH2, n=1656 genes) using *enrich* tool. **b**, Pathways commonly represented in ERG and mERG/EZH2 GS. **c**, Heat Map using the GS_mERG/EZH2 set applied to primary prostate tumors (Primary) and castration-resistant prostate cancers (CRPC) with known ERG and EZH2 status in the Michigan dataset. Color bars indicate ERG and EZH2 expression status.

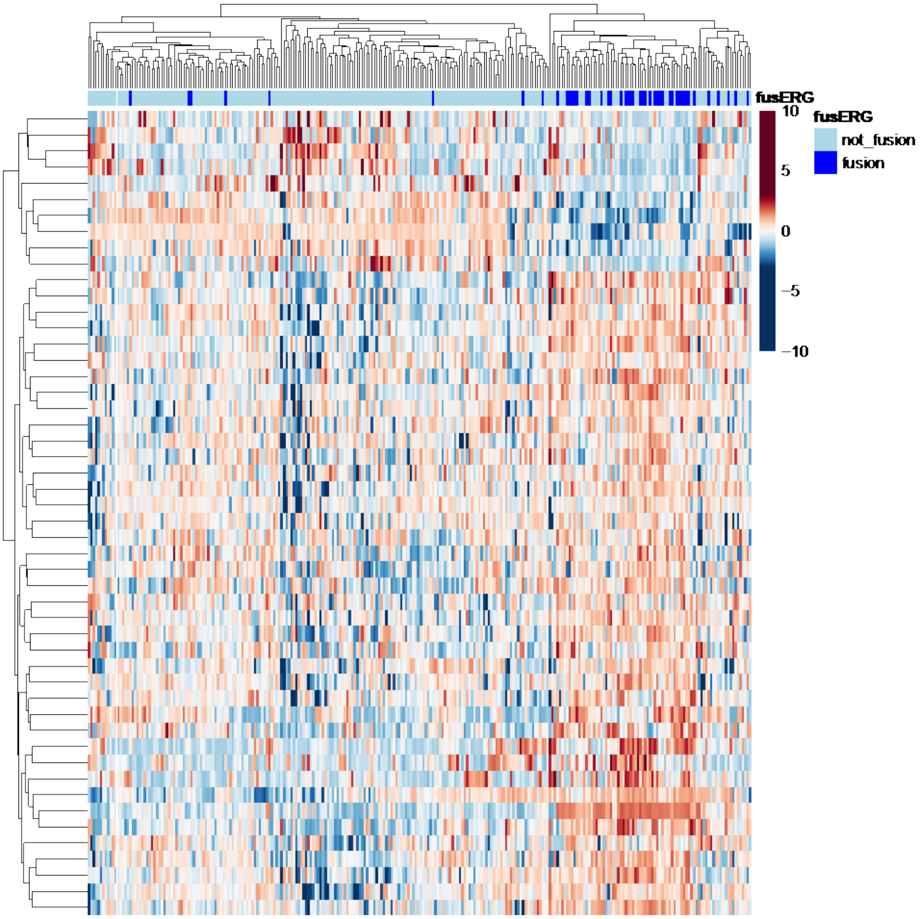

Sboner dataset _HM (GS_ERG/EZH2_ 50)

Supplementary Figure 9. ERG/EZH2 co-regulated genes are expressed in aggressive prostate tumors.

HM with the top deregulated ERG/EZH2 gene set (GS_ERG/EZH2_50) in the Sboner dataset. Color bars indicate fusion ERG status.

Supplementary Table 1:
siRNAs and shRNA information

siGL3	5’ CUUACGCUGAGUACUUCGAtt 3’<br>5’ UCGAAGUACUCAGCGUAAGtt 3’
siRNA Negative Control (NC) for DLA	Catalog n. 4611 (Ambion)
siERG	Catalog n. SI03089443 (Qiagen)
siEZH2	5’ CAAAGAAUCUAGCAUCAUAtt 3’<br>5’ UAUGAUGCUAGAUUCUUUGtt 3’
siEZH2 3UTR	5’ GCAAUUUAGAAAAAGAACAtt 3’<br>5’ UGUUCUUUUUCUAAAUUGCcc 3’
siSUZ12	5’ GGAUGUAAGUUGUCCAAUAtt 3’<br>5’UAUUGGACAACUUACAUCCtt 3’
siPTEN	5’ GCAUACGAUUUUAAGCGGAtt 3’<br>5’ UCCGCUUAAAAUCGUAUGCag 3’
shPTEN	Clone ID:NM_000314.4-2996s21c1 (Sigma)
siERG#1	Catalog n. 4392420 assay i.d. s4812 (Thermofisher Scientific)
siERG#2	Catalog n. 4392420 assay i.d. s4813 (Thermofisher Scientific)
shERG#1	cat number TRCN0000429354 Clone ID: NM_004449.4-331s21c1 SIGMA
shERG#2	cat number TRCN0000429394 Clone ID: NM_004449.4-1642s21c SIGMA

Supplementary Table 2: Primer sets used for RT-PCR, ChIP and mutagenesis.

Gene ID	Assay	Forward (FWD) and reverse (REV) primer sequences
human IL-6	qRT-PCR	FWD: CCACACAGACAGCCACTCAC
human IL-6	qRT-PCR	REV: TTTCAGCCATCTTTGGAAGG
human NKX 3.1	qRT-PCR	FWD: AGAAAGGCACTTGGGGTCTT
human NKX 3.1	qRT-PCR	REV: TCCTCTCCAACTCGATCACC
human ARID2	qRT-PCR	FWD: GAAGGAGCTGGATCTTCACG
human ARID2	qRT-PCR	REV: AAGCAAAGGCAGCGTTAGAA
human BMI1	qRT-PCR	FWD: TTCTTTGACCAGAACAGATTGG
human BMI1	qRT-PCR	REV: GCATCACAGTCATTGCTGCT
human CADPS	qRT-PCR	FWD: CCGAATGGATAAGCCTCAAA
human CADPS	qRT-PCR	REV: ATAACTGCACATGGCAAACG
human CTNNA1	qRT-PCR	FWD: CATGTTTTGGCTGCATCTGT
human CTNNA1	qRT-PCR	REV: CAGCAGCCTTCATCAAATCA
human DAAM1	qRT-PCR	FWD: TGCACTTCCAGCTGAGAAAA
human DAAM1	qRT-PCR	REV: TCAGTGCTGTCTTTAAACTCTCT
human MAT2A	qRT-PCR	FWD: AGGGATGCCCTAAAGGAGAA
human MAT2A	qRT-PCR	REV: ATTTTGCGTCCAGTCAAACC
mouse Rn18s	qRT-PCR	FWD: ACCGCAGCTAGGAATAATG
mouse Rn18s	qRT-PCR	REV: GCCTCAGTTCCGAAAACCA
mouse IL-6	qRT-PCR	FWD:TCCTTCCTACCCCAATTTCC
mouse IL-6	qRT-PCR	REV: GCACTAGGTTTGCCGAGTAGAT
mouse ARID2	qRT-PCR	FWD: TCATGCAGCTTGAGAAGGATCC
mouse ARID2	qRT-PCR	REV: AGAAGAGTGGCGAGAGAAGACT
mouse BMI1	qRT-PCR	FWD: GAAATGGCCCACTACCTTTG
mouse BMI1	qRT-PCR	REV: CTTTCCAGCTCTCCAGCATT
mouse CADPS	qRT-PCR	FWD: CTCAGAAGCATGGCATGGATGA
mouse CADPS	qRT-PCR	REV: GGCTGACTTTGGATCACAGACT
mouse CTNNA1	qRT-PCR	FWD: AGGCCGTCACAGGAATTTCT
mouse CTNNA1	qRT-PCR	REV: ATTGTGGACCCCTTGAGCTT
mouse DAAM1	qRT-PCR	FWD: AACCCGCTTCCAGACGTTAA
mouse DAAM1	qRT-PCR	REV: ATCAATGCAGTGCTGAGCCA
mouse MAT2A	qRT-PCR	FWD: GAGGGTTCTTGTTCAGGTCTC
mouse MAT2A	qRT-PCR	REV: GAATTTTGATCTTCGCCCTGGG
mouse EZH2	qRT-PCR	FWD: CATCCCGTTAAAGACCCTGA
mouse EZH2	qRT-PCR	REV: CCAGAACTTCATCCCCCATA
mouse Lin28A	qRT-PCR	FWD: TAAGAAGTCTGCCAAGGGTCTG
mouse Lin28A	qRT-PCR	REV: CCTTTGGATCTTCGCTTCTG
mouse POU5F1	qRT-PCR	FWD: CAAGTTGGCGTGGAGACTTT
mouse POU5F1	qRT-PCR	REV: TTGGTTCCACCTTCTCCAAC
mouse IL-6 promoter	ChIP	FWD: AGGAGTGTGAGGCAGAGAGC
mouse IL-6 promoter	ChIP	REV: AGTCTCCTGCGTGGAGAAAA
mouse ARID2 promoter	ChIP	FWD: AAGCACGCTCCGCTCTATTA
mouse ARID2 promoter	ChIP	REV: TAGTCTTTCCGATGGGAGCA
mouse BMI1 promoter	ChIP	FWD: CTCGGTGCCCATTGACAG
mouse BMI1 promoter	ChIP	REV: AATGAATGCGAGCCAAGC
mouse MAT2A promoter	ChIP	FWD: CCCTTCTCGATTTCTGGAAC
mouse MAT2A promoter	ChIP	REV: CCTTTCTCCGCTCCTCATC
human IL-6 promoter EBS	ChIP	FWD: ACAGCTGGGAAGACGAGAAA
human IL-6 promoter EBS	ChIP	REV: GGAAGTTCGTGTTCATGATAAAA
human NKX 3.1 promoter EBS	ChIP	FWD: TGCGGATAAAGGAACCACCA
human NKX 3.1 promoter EBS	ChIP	REV: AGGCATGACAAGTAGGTGCAGC
human NKX 3.1 promoter ARE	ChIP	FWD: TCGCGGTGAGAAAATCAGTGT

human NKX 3.1 promoter ARE	ChIP	REV: TGAAAAGCATGCCCTGGTG
human ARID2 promoter	ChIP	FWD: ACATCGCCTCCACCCCTA
human ARID2 promoter	ChIP	REV: CGGGGAACAATAGACTCGAC
human BMI1 promoter	ChIP	FWD: GGCTCGCATTCATTTTCTGC
human BMI1 promoter	ChIP	REV: GCCTCGCCTCCTACGTAC
human CADPS promoter	ChIP	FWD: CTCCCCAATCGGCAAGAT
human CADPS promoter	ChIP	REV: GGCAAGGGGGAGAATCAAT
human CTNNA1 promoter	ChIP	FWD: AGAATGACATGGGGAACAGC
human CTNNA1 promoter	ChIP	REV: TAAGCACGTGGTGATTCAGG
human DAAM1 promoter	ChIP	FWD: ACAAACTAGGTCGGCAGGAA
human DAAM1 promoter	ChIP	REV: GGACAGAGAACACACACCCT
human MAT2A promoter	ChIP	FWD: TTGGGAATGCACCTTGTTCT
human MAT2A promoter	ChIP	REV: ACAAGAACGCCGGGTTTAAT
ΔC-ERG3	Mut.	CGCCTACAAGTTCGACTAACACGGGATCGCCCAGG
P-I-ERG3	Mut.	CAGGCAGTGGCCAGATCTTAACTTTGGCAGTTCCTC CTGG
P-ERG3	Mut.	CCTCAGAGAGACTCCTCTTCCATAATTGACTTCAGAT GATGTTG
N-ERG3	Mut.	GCTACATGGAGGAGAAGCACTAACCACCCCCAAACA TGACC
ERG-K362A	Mut.	CCGGCGCTGGGGAGAGCGGGCGAGCAAAC CCAACATG
EZH2-ΔCXC	Mut.	CAGCTGTATCTTTCTCTAGTGTGCAGCCCAC AACCGG
EZH2-1-340	Mut.	GCTGCTGCTCTCACCGCTTAGCGGATAAAG ACCCCACC
S21A-EZH2	Mut.	GTTGGCGGAAGCGTGTAAAAGCAGAGTACATGCGA CTGAGACAG
S21D-EZH2	Mut.	GTTGGCGGAAGCGTGTAAAAGACGAGTACATGCGA CTGAGACAG
