# Supplementary material for: EZH2-induced lysine K362 methylation enhances TMPRSS2-ERG oncogenic activity in prostate cancer
Source: Nat Commun. 2021 Jul 6;12:4147. doi: 10.1038/s41467-021-24380-6 (PMC8260656; doi:10.1038/s41467-021-24380-6)
Supplement: Supplementary file 2 — Description of Additional Supplementary Files [file 41467_2021_24380_MOESM2_ESM.pdf]

Description of Additional Supplementary Files

Supplementary data 1

Description: Genes differentially modulated in LNCAP cells stably transfected with ERG wild type (ERGWT),ERG K362 mutant (ERGK362A) or empty vector (EV).Lists include gene upregulated and downregulated in ERGWT vs EV and ERGK362A vs EV using logfold change values (logFC).

Supplementary data 2

Description: ERG_EZH2 targets deregulated (enhanced and attenuated) in castration resistant prostate tumors (CRPC) versus Primary prostate tumors (PR) in the Michigan database (cBioPortal: Michigan, Nature 2012 - GSE35988), shown in Figure 8c.

Supplementary data 3

Description: mERG_EZH2 targets deregulated (enhanced and attenuated) in CRPC versus Primary prostate tumors (PR) in the Michigan database (cBioPortal: Michigan, Nature 2012 - GSE35988), shown in Supplementary Figure 8c.
