# Supplementary material for: EZH2-induced lysine K362 methylation enhances TMPRSS2-ERG oncogenic activity in prostate cancer
Source: Nat Commun. 2021 Jul 6;12:4147. doi: 10.1038/s41467-021-24380-6 (PMC8260656; doi:10.1038/s41467-021-24380-6)
Supplement: Supplementary file 6 — Reporting Summary [file 41467_2021_24380_MOESM6_ESM.pdf]

nature research

Corresponding author(s): Giuseppina M Carbone

Last updated by author(s): APRIL 21 2021

Reporting Summary

Nature Research wishes to improve the reproducibility of the work that we publish. This form provides structure for consistency and transparency in reporting. For further information on Nature Research policies, see our Editorial Policies and the Editorial Policy Checklist.

Statistics

For all statistical analyses, confirm that the following items are present in the figure legend, table legend, main text, or Methods section.

n/a	Confirmed	
☐	☒	The exact sample size (*n*) for each experimental group/condition, given as a discrete number and unit of measurement
☐	☒	A statement on whether measurements were taken from distinct samples or whether the same sample was measured repeatedly
☐	☒	The statistical test(s) used AND whether they are one- or two-sided<br>*Only common tests should be described solely by name; describe more complex techniques in the Methods section.*
☐	☒	A description of all covariates tested
☐	☒	A description of any assumptions or corrections, such as tests of normality and adjustment for multiple comparisons
☐	☒	A full description of the statistical parameters including central tendency (e.g. means) or other basic estimates (e.g. regression coefficient) AND variation (e.g. standard deviation) or associated estimates of uncertainty (e.g. confidence intervals)
☐	☒	For null hypothesis testing, the test statistic (e.g. *F*, *t*, *r*) with confidence intervals, effect sizes, degrees of freedom and *P* value noted<br>*Give P values as exact values whenever suitable.*
☒	☐	For Bayesian analysis, information on the choice of priors and Markov chain Monte Carlo settings
☐	☒	For hierarchical and complex designs, identification of the appropriate level for tests and full reporting of outcomes
☒	☐	Estimates of effect sizes (e.g. Cohen's *d*, Pearson's *r*), indicating how they were calculated

Our web collection on statistics for biologists contains articles on many of the points above.

Software and code

Policy information about availability of computer code

Data collection	Data were collected by using Microsoft Excel 2016 64-bit (Microsoft), IMAGEJ version 1.51c (NIH:https://imagej.nih.gov/ij/), Zeiss Microscope with Canon EOS 450D camera, ImageScope Slide Viewing APERIO Software 12.1. GraphPad Prism Version 6, Version 7 (GraphPad), StepOne software v2.2 (Life Technologies), FUSION-Capt software (Fusion Solo 7S: Witec), Living Image software 4.2 (IVIS Imaging Systems: Caliper LifeSciences), Amber (University of California, San Francisco, Version: 16), Gromacs (www.gromacs.org, Version: 4.6), VMD - Visual Molecular Dynamics (version 1.9.4), StepOne software version 2.2 (Life Technologies).
Data analysis	For the ChipSeq analysis, the following tool were exploited: bowtie (version 2.3.4.2), MACS14, ChIPpeakAnno_3.22.4. For the further analysis on ChipSeq data deepTools, MEME tool, and Venn diagram generator tool were used.<br>The differential expression analysis in microarray and RNA-seq databases were performed through limma_3.44.3 and DESeq2_1.28.1 R packages, respectively. For the other downstream analyses, the following R packages were used: DOSE_3.14.0, PCAtools_2.0.0, pheatmap_1.0.12. Additional tools included BRB-ArrayTools, Enrichr (http://amp.pharm.mssm.edu/Enrichr/), GSEA from the Broad Institute (http://www.broadinstitute.org/gsea/). Shake Algorithm (Journal of Computational Physics. 1977;23(3):327–341).

For manuscripts utilizing custom algorithms or software that are central to the research but not yet described in published literature, software must be made available to editors and reviewers. We strongly encourage code deposition in a community repository (e.g. GitHub). See the Nature Research guidelines for submitting code & software for further information.

Data

Policy information about availability of data

All manuscripts must include a data availability statement. This statement should provide the following information, where applicable:

- Accession codes, unique identifiers, or web links for publicly available datasets
- A list of figures that have associated raw data
- A description of any restrictions on data availability

The used ChipSeq datasets are: GSE14097 (human), GSE28951 (VCaP cells), GSE159471 (in house mERG). The databases of transcriptomic data that were used are: Sboner dataset, Weill Cornell Medical College (GSE16560); TCGA dataset (downloaded from http://gdac.broadinstitute.org/); the Prostate Adenocarcinoma dataset, (cBioPortal: Michigan, Nature 2012 - GSE35988).

Field-specific reporting

Please select the one below that is the best fit for your research. If you are not sure, read the appropriate sections before making your selection.

[x] Life sciences [ ] Behavioural & social sciences [ ] Ecological, evolutionary & environmental sciences

For a reference copy of the document with all sections, see nature.com/documents/nr-reporting-summary-flat.pdf

Life sciences study design

All studies must disclose on these points even when the disclosure is negative.

Sample size	Sample sizes were estimated from other experiments obtained by similar experiments performed in former publications of the group. (Citations listed below)<br>In vitro studies: Sample sizes were determined based on pilot studies results and on previous similar studies from this group.<br>In vivo studies: Animal experiments were conducted by using between 3 and 6 mice per group. This sample sizes in previously and similar studies, have given statistical significant results based on the variance of xenografts growth in control mice. For ethical reasons, the minimum number of animals necessary to achieve the scientific objectives was used.<br><br>Citations:<br>https://doi.org/10.1038/s42003-020-01642-5<br>https://doi.org/10.1016/j.euo.2019.07.013<br>https://doi.org/10.1016/j.cmet.2019.05.004<br>https://doi.org/10.1016/j.euo.2018.08.024<br>https://doi.org/10.3389/fonc.2019.00385<br>https://doi.org/10.18632/oncotarget.12525<br>https://doi.org/10.1016/j.molonc.2015.02.012<br>https://doi.org/10.1038/sj.onc.1210953
Data exclusions	No data excluded
Replication	All experiments were performed with sufficient biological and technical replicates with cells and animals per group in order to demonstrate statistical significance. Number of replicates in each experiment is indicated in the corresponding figure or legend.
Randomization	For in vivo experiments animals were randomly subdivided into respective groups for experiments. For in vitro and other experiments no randomization was necessary.
Blinding	For experiments blinding was not necessary during the experimentation and data collection. As the results were quantitative and did not require critical interpretations or subjective judgment. Also these groups were well defined and studied using standard protocols.

Reporting for specific materials, systems and methods

We require information from authors about some types of materials, experimental systems and methods used in many studies. Here, indicate whether each material, system or method listed is relevant to your study. If you are not sure if a list item applies to your research, read the appropriate section before selecting a response.

Materials & experimental systems

n/a	Involved in the study
	☒ Antibodies
	☒ Eukaryotic cell lines
☒	☐ Palaeontology and archaeology
	☒ Animals and other organisms
	☒ Human research participants
☒	☐ Clinical data
☒	☐ Dual use research of concern

Methods

n/a	Involved in the study
	☒ ChIP-seq
☒	☐ Flow cytometry
☒	☐ MRI-based neuroimaging

Antibodies

Antibodies used

Rabbit polyclonal anti-mERG (AbMart: custom made)
Rabbit polyclonal anti-ERG (Santa Cruz: sc-353)
Mouse monoclonal anti-ERG (Santa Cruz: sc-271048)
Rabbit polyclonal anti-ERG (Santa Cruz: sc-354)
Rabbit monoclonal anti-ERG (Abcam: ab92513)
Rabbit monoclonal anti-ERG (Epitomics: 2805-1)
Rabbit monoclonal anti-EZH2 (Cell Signaling: #5246)
Mouse monoclonal anti-EZH2 (BD Biosciences: 612667)
Rabbit polyclonal anti-pS21 EZH2 (Bethyl Laboratories: IHC-00388)
Mouse monoclonal anti-EZH2 (Active Motif: 39875, 39876)
Rabbit polyclonal anti-PTEN (Cell Signaling: #9552)
Mouse monoclonal anti-pAkt (Cell Signaling: #4051)
Rabbit polyclonal anti-Akt (Cell Signaling : #9272)
Rabbit polyclonal anti-AR (Millipore: #06-680)
Mouse monoclonal anti-HA (Santa Cruz: sc-7392)
Mouse monoclonal Anti-polyHistidine (Sigma Aldrich: h1029)
Rabbit polyclonal anti-Methylated Lysine (Abcam: ab23366)
Rabbit monoclonal Tri-Methyl-Histone H3 (Lys27) (Cell Signaling: #9733)
Rabbit Monoclonal anti Ki67 (Lab Vision: RM-9106-R7)
Rabbit anti-histone H3 acetyl (Active Motif: 39139, 39140)
Rabbit polyclonal anti-EED (Millipore: #09-774)
Rabbit polyclonal anti-SUZ12 (AbCam: ab12073)
Rabbit polyclonal anti-SUZ12 (Active Motif: 39357, 39358)
Normal Mouse IgG (Millipore: 12-371)
anti-GAPDH (Santa Cruz: sc-47724)
anti-α-tubulin (Calbiochem: Cp06)
Goat polyclonal anti-β-Actin (Santa Cruz: sc-1616)
Rabbit monoclonal anti-HSP90 (Cell Signaling: #4877)
R.T.U Biotinilated anti rabbit (Vector Lab: BP-9100)
Alexa Fluor 488 anti-Rabbit (Invitrogen A-11008)
Alexa Fluor 594 anti-Rabbit (ThermoFisher Scientific A-11012)

Validation

Rabbit polyclonal anti-mERG (AbMart: custom made)
Validation data included in the paper.

Rabbit polyclonal anti-ERG (Santa Cruz: sc-353)
Validation: https://www.citeab.com/antibodies/791921-sc-353-erg-1-2-3-c-20

Mouse monoclonal anti-ERG (Santa Cruz: sc-271048)
Validation: https://www.scbt.com/p/erg-1-2-3-antibody-d-3

Rabbit polyclonal anti-ERG (Santa Cruz: sc-354)
Validation: https://www.scbt.com/p/erg-1-2-3-antibody-c-17

Rabbit monoclonal anti-ERG (Abcam: ab92513)
Validation: https://www.abcam.com/erg-antibody-epr3864-ab92513.html

Rabbit monoclonal anti-ERG (Epitomics: 2805-1)
Validation: https://www.citeab.com/antibodies/2868645-2805-1-erg-rabmab

Rabbit monoclonal anti-EZH2 (Cell Signaling: #5246)
Validation: https://www.cellsignal.com/products/primary-antibodies/ezh2-d2c9-xp-rabbit-mab/5246

Mouse monoclonal anti-EZH2 (BD Biosciences: 612667)
Validation: https://www.bdbiosciences.com/us/applications/research/stem-cell-research/cancer-research/human/purified-mouse-anti-ezh2-11ezh2/p/612667

Rabbit polyclonal anti-pS21 EZH2 (Bethyl Laboratories: IHC-00388)
Validation: https://www.citeab.com/antibodies/1039049-ihc-00388-phospho-ezh2-s21-ihc-antibody

Mouse monoclonal anti-EZH2 (Active Motif: 39875, 39876)
Validation: https://www.activemotif.com/catalog/details/39875/ezh2-antibody-mab-clone-ac22

Rabbit polyclonal anti-PTEN (Cell Signaling: #9552)
Validation: https://www.cellsignal.com/products/primary-antibodies/pten-antibody/9552

Mouse monoclonal anti-pAkt (Cell Signaling: #4051)
Validation: https://www.cellsignal.co.uk/products/primary-antibodies/phospho-akt-ser473-587f11-mouse-mab/4051

Rabbit polyclonal anti-Akt (Cell Signaling: #9272)
Validation: https://www.cellsignal.com/products/primary-antibodies/akt-antibody/9272

Rabbit polyclonal anti-AR (Millipore: #06-680)
Validation: https://www.merckmillipore.com/CH/de/product/Anti-Androgen-Receptor-Antibody,MM_NF-06-680

Mouse monoclonal anti-HA (Santa Cruz: sc-7392)
Validation: https://www.scbt.com/p/ha-probe-antibody-f-7

Mouse monoclonal Anti-polyHistidine (Sigma Aldrich: h1029)
Validation: https://www.sigmaaldrich.com/catalog/product/sigma/h1029?lang=fr&region=CH

Rabbit polyclonal anti-Methylated Lysine (Abcam: ab23366)
Validation: https://www.abcam.com/methylated-lysine-di-methyl--mono-methyl--antibody-ab23366.html

Rabbit monoclonal Tri-Methyl-Histone H3 (Lys27) (Cell Signaling: #9733)
Validation: https://www.cellsignal.com/products/primary-antibodies/tri-methyl-histone-h3-lys27-c36b11-rabbit-mab/9733

Rabbit Monoclonal anti Ki67 (Lab Vision: RM-9106-R7)
Validation: https://assets.thermofisher.com/TFS-Assets/APD/Specification-Sheets/D12537~.pdf

Rabbit anti-histone H3 acetyl (Active Motif: 39139, 39140)
Validation: https://www.activemotif.com/catalog/details/39139/histone-h3ac-pan-acetyl-antibody-pab-1

Rabbit polyclonal anti-EED (Millipore: #09-774)
Validation: https://www.merckmillipore.com/CH/de/product/Anti-EED-Antibody,MM_NF-09-774

Rabbit polyclonal anti-SUZ12 (AbCam: ab12073)
Validation: https://www.abcam.com/suz12-antibody-ab12073.html

Rabbit polyclonal anti-SUZ12 (Active Motif: 39357, 39358)
Validation: https://www.activemotif.com/catalog/details/39357/suz12-antibody-pab

Normal Mouse IgG (Millipore: 12-371)
Validation: https://www.merckmillipore.com/CH/de/product/Normal-Mouse-IgG,MM_NF-12-371

anti-GAPDH (Santa Cruz: sc-47724)
Validation: https://www.scbt.com/p/gapdh-antibody-0411

anti-α-tubulin (Calbiochem: Cp06)
Validation: https://www.merckmillipore.com/CH/de/product/Anti-Tubulin-Mouse-mAb-DM1A,EMD_BIO-CP06

Goat polyclonal anti-β-Actin (Santa Cruz: sc-1616)
Validation: https://www.scbt.com/p/actin-antibody-i-19

Rabbit monoclonal anti-HSP90 (Cell Signaling: #4877)
Validation: https://www.cellsignal.com/products/primary-antibodies/hsp90-c45g5-rabbit-mab/4877

R.T.U Biotinilated anti rabbit (Vector Lab: BP-9100)
Validation: https://vectorlabs.com/rtu-biotinylated-goat-anti-rabbit-igg-antibody.html

Alexa Fluor 488 anti-Rabbit (Invitrogen A-11008)

Validation: https://www.thermofisher.com/order/genome-database/dataSheetPdf?producttype=antibody&productsubtype=antibody_secondary&productId=A-11008&version=137

Alexa Fluor 594 anti-Rabbit (ThermoFisher Scientific A-11012)
Validation: https://www.thermofisher.com/order/genome-database/generatePdf?productName=Rabbit%20IgG%20(H+L)&assayType=PRANT&productId=A-11012&detailed=false

Eukaryotic cell lines

Policy information about cell lines

Cell line source(s)	VCaP (ATCC: CRL-2876)<br>PC3 (ATCC: CRL-1435)<br>LNCaP (ATCC: CRL-1740)<br>RWPE1 (ATCC: CRL-11609)
Authentication	All cell lines used were purchased from ATCC. ATCC uses PCR based approaches, karyotyping, and morphology to confirm the identity of human cell lines.
Mycoplasma contamination	All cell lines were tested for mycoplasma contamination periodically, via the MycoAlert Mycoplamsa Testing kit (Lonza). Results were always negative for mycoplasma contamination.
Commonly misidentified lines (See ICLAC register)	No commonly misidentified cell lines were used in present study.

Animals and other organisms

Policy information about studies involving animals; ARRIVE guidelines recommended for reporting animal research

Laboratory animals	Male of NOD.Cg-PrkdcSCID Il2rgtm1Wjl/SzJ (NSG-KO) mice (4–6 weeks old, Jackson Laboratories) were used for xenografts establishment. The PbCre4; Pten flox/+ R26LSL;ERG male mice (24–26 weeks old) were used in the experiments (Line generously provided by Dr. Charles L. Sawyers). Cages were well ventilated, softly lit and subject to a light/dark cycle. The relative humidity kept at 45 to 65%. Mouse rooms and cages kept at a temperature range of 20-24C.
Wild animals	No wild animals were used.
Field-collected samples	No field-collected samples were used.
Ethics oversight	All mouse studies were approved by the MSKCC Institutional Animal Care and Use Committee under protocol 06-07-012.

Note that full information on the approval of the study protocol must also be provided in the manuscript.

Human research participants

Policy information about studies involving human research participants

Population characteristics	Age ranges of individuals with cancer prostate were 50–74 years. Clinical parameters such as Gleason, tumor stage, and PSA values were recorded at the time of surgery.
Recruitment	Tumor samples were taken from patients undergone to radical prostatectomy.
Ethics oversight	Tissue samples were collected with the approval of the Ethics Committee of the Piedmont Region, Italy, and patients' written informed consent.

Note that full information on the approval of the study protocol must also be provided in the manuscript.

ChIP-seq

Data deposition

☒ Confirm that both raw and final processed data have been deposited in a public database such as GEO.

☒ Confirm that you have deposited or provided access to graph files (e.g. BED files) for the called peaks.

Data access links<br>*May remain private before publication.*	https://urldefense.proofpoint.com/v2/url?u=https-3A__www.ncbi.nlm.nih.gov_geo_query_acc.cgi-3Facc-3DGSE159471&d=DwIBAg&c=5rLNXN0mp_7LMh3Fds96xpjyD06ZuE2RU7zikolS0lg&r=Y9I0rPapB9uKro-rSQB13od67VlLQ9x3ejJIU_fa3ZmfBHVJsGwNS3LX-sRf-pZf&m=PmoB5_JAdaOxl_urwtIPvRryUOSkWKRuolFJ-g286rk&s=UoEJAdclLBdl42iXFgSlKqktTM_gl1chTUNYyq800qk&e=
Files in database submission	(1) GPL18573 Illumina NextSeq 500 (Homo sapiens)

	(2) GSM4830211 VCaP mERG<br>(3) GSM4830212 VCaP INPUT
Genome browser session<br>(e.g. UCSC)	No longer applicable.

Methodology

Replicates	For in-house Chip: 1 replicate per condition (Control input, Chip mERG)
Sequencing depth	Total number of reads: 38344564; read length: 75; single-end reads
Antibodies	Rabbit polyclonal anti-mERG (AbMart: custom made)<br>Validation data included in the manuscript.
Peak calling parameters	macs14 -t Chip.bam -c INPUT.bam --name=macs --gsize=2.72e9 --format=BAM --wig --single-profile --pvalue=0.005 --format=BAM --space=10
Data quality	The data quality was ensured with FastQC
Software	For the ChipSeq analysis, the following tool were exploited: bowtie, MACS14, ChIPpeakAnno_3.22.4
